# The crystal structure of KSHV ORF57 reveals dimeric active sites important for protein stability and function

**DOI:** 10.1371/journal.ppat.1007232

**Published:** 2018-08-10

**Authors:** Fei Yuan, Zeng-Qiang Gao, Vladimir Majerciak, Lei Bai, Meng-Lu Hu, Xiao-Xi Lin, Zhi-Ming Zheng, Yu-Hui Dong, Ke Lan

**Affiliations:** 1 State Key Laboratory of Virology, College of Life Sciences, Medical Research Institute, Wuhan University, Wuhan, P. R. China; 2 Beijing Synchrotron Radiation Facility, Institute of High Energy Physics, Chinese Academy of Sciences, Beijing, China; 3 Tumor Virus RNA Biology Section, RNA Biology Laboratory, Center for Cancer Research, National Cancer Institute, National Institutes of Health, Frederick, Maryland, United States of America; University of Southern California, UNITED STATES

## Abstract

Kaposi’s sarcoma-associated herpesvirus (KSHV) is a γ-herpesvirus closely associated with Kaposi’s sarcoma, primary effusion lymphoma and multicentric Castleman disease. Open reading frame 57 (ORF57), a viral early protein of KSHV promotes splicing, stability and translation of viral mRNA and is essential for viral lytic replication. Previous studies demonstrated that dimerization of ORF57 stabilizes the protein, which is critical for its function. However, the detailed structural basis of dimerization was not elucidated. In this study, we report the crystal structures of the C-terminal domain (CTD) of ORF57 (ORF57-CTD) in both dimer at 3.5 Å and monomer at 3.0 Å. Both structures reveal that ORF57-CTD binds a single zinc ion through the consensus zinc-binding motif at the bottom of each monomer. In addition, the N-terminal residues 167–222 of ORF57-CTD protrudes a long “arm” and holds the globular domains of the neighboring monomer, while the C-terminal residues 445–454 are locked into the globular domain *in cis* and the globular domains interact *in trans*. *In vitro* crosslinking and nuclear translocation assays showed that either deletion of the “arm” region or substitution of key residues at the globular interface led to severe dimer dissociation. Introduction of point mutation into the zinc-binding motif also led to sharp degradation of KSHV ORF57 and other herpesvirus homologues. These data indicate that the “arm” region, the residues at the globular interface and the zinc-binding motif are all equally important in ORF57 protein dimerization and stability. Consistently, KSHV recombinant virus with the disrupted zinc-binding motif by point mutation exhibited a significant reduction in the RNA level of ORF57 downstream genes ORF59 and K8.1 and infectious virus production. Taken together, this study illustrates the first structure of KSHV ORF57-CTD and provides new insights into the understanding of ORF57 protein dimerization and stability, which would shed light on the potential design of novel therapeutics against KSHV infection and related diseases.

## Author summary

KSHV encoded multifunctional protein ORF57 plays a key role in promoting viral mRNA accumulation and translation. Previous biochemical studies showed that ORF57 is stabilized by dimerization, which is critical for its function. To better understand ORF57 dimerization, we resolved the structure of the C-terminal domain (CTD) of ORF57. We found that ORF57-CTD interacts with the neighboring monomer through the “arm” region and the globular interface, and demonstrated that they are responsible for dimerization. In addition, we identified a zinc ion chelated by four residues of a zinc-binding motif within ORF57-CTD and this motif is conserved among herpesvirus homologues, participating in protein folding and stability. By defining the first crystal structure of ORF57-CTD, this study reveals the detailed mechanisms of ORF57 stability and dimerization, and provides novel insights for therapeutic development against KSHV-related diseases.

## Introduction

Kaposi’s sarcoma-associated herpesvirus (KSHV, also known as human herpesvirus 8, HHV8) is a human tumor virus belonging to lymphotropic gammaherpesvirus subfamily [1]. KSHV was first discovered from Kaposi’s sarcoma with endothelial origin, and also found as the etiological agent of two lymphoproliferative disorders, primary effusion lymphoma (PEL) and multicentric Castleman’s disease (MCD) [2–4]. Like other herpesviruses, KSHV establishes life-long persistent latent infection. Endogenous and exogenous stress can trigger KSHV reactivation [5] and viral gene expression in a cascade fashion. During reactivation, the viral lytic switch protein RTA (replication and transcription activator) activates the expression of viral early genes including MTA (mRNA transcript accumulation) encoded by open reading frame 57 (ORF57) [6]. ORF57 or MTA translocates into cell nucleus and promotes lytic replication [7]. Thus, ORF57 deletion virus leads to defective expression of viral lytic genes and abortive viral replication, suggesting that ORF57 is indispensable for KSHV lytic replication [8–10].

During lytic replication, ORF57 promotes different viral mRNA accumulation and gene expression through distinct mechanisms. ORF57 stabilizes polyadenylated nuclear (PAN) RNA by cooperative binding to a 9-nt core of the MRE (MTA responsive element) within PAN RNA with PABPC1 [11]. ORF57 also enhances ORF59 mRNA stabilization through preventing RBM15-mediated hyperpolyadenylation and nuclear retention [12,13]. Moreover, ORF57 directly interacts with SRSF3, thereby preventing SRSF3 from binding to K8β intron and increasing K8β splicing [14]. ORF57 also binds to the MTA responsive element (MRE) of vIL-6 to prevent the miR-1293–Ago2 RISC from associating with vIL-6 RNA and enhance vIL-6 translation [15]. Recent report shows ORF57 interaction with PACT and PKR and to antagonize host antiviral defenses for virus lytic infection and production[16]. In addition, ORF57 might sequester hTREX complex to promote double-strand break response and DNA damage [17].

As an important viral protein, the domain organization and the functional elements of ORF57 has been extensively studied [18]. ORF57 comprises of two domains with distinct putative structures: the highly disordered N-terminal domain (NTD) from residue 1 to 166, and the helix-rich C-terminal domain (CTD) from residue 167 to 455. Nuclear localization of ORF57 is mediated by the three nuclear localization signals (NLSs) within NTD [7]. The flexible NTD facilitates binding diversity and interacts with a number of cellular factors [19]. NTD is highly phosphorylated, and the phosphorylation sites include S95/S97 identified by mass spectrometry and S21/T32/S43 identified by *in vitro* phosphorylation assay [18]. NTD also contains a caspase-7 cleavage site as a cellular antiviral response, and blockade of caspase-7 cleavage promotes the expression of a subset of viral lytic genes [20]. In contrast to the NTD of which the protein sequence is not highly conserved, CTD is relatively conserved in the putative secondary structure among herpesvirus homologues, including ICP27 of HSV1 (Herpes Simplex Virus Type 1) [21,22], UL69 of HCMV (Human Cytomegalovirus) [23], IE4 of VZV (Varicella-Zoster Virus) [24], EB2 of EBV (Epstein-Barr Virus) [25] and mORF57 of MHV68 (Murine Gamma Herpesvirus 68). The CTD of KSHV ORF57 mediates self-interaction, which is a common feature found in other homologues [21,22,26,27]. So far, only the structure of the C-terminal HSV1 ICP27 (aa 241–512) [21,22] and solution structure of partial HVS ORF57 (aa 103–120)-ALYREF (aa 54–155) complex have been resolved [28].

Despite extensive studies on the important functions of ORF57 during KSHV lytic replication, the structural basis for the characterized ORF57 functions remains unclear. In the previous study, Majerciak et al. identified three dimerization-essential residues E287, E288 and W292 by synthetic peptide competition assays and point mutations and demonstrated that ORF57-CTD dimerization prevents protein degradation [18]. However, the structural relationship between ORF57 dimerization and stability was not fully explored. In this study, we resolved the crystal structures of ORF57-CTD in dimer at 3.5 Å and in monomer at 3.0 Å by X-ray diffraction. Each monomer CTD has an N-terminal arm and a C-terminal globular domain bearing a zinc-binding motif. The arm from one monomer hooks the globular domain of a neighboring monomer in an anti-parallel manner and makes the aligned globular domain residues expose to each other. The electrostatic interactions of the interface residues between two anti-parallel monomers further stabilize the dimerization. Functional studies demonstrated that deletion or point mutation of either one of three structural elements (zinc-binding motif, arm and globular interface) prevents ORF57 from dimerization and destabilizes ORF57.

## Results

### Protein purification, crystallization, dehydration and data collection

ORF57-CTD (from residue 167 to 455) is a theoretical 32 kDa protein, with an isoelectric point (pI) approximately 8.5. Previous studies indicated that the majority of ORF57-CTD exists as dimers [18]. Expression and purification processes have been described in experimental procedures.

Two kinds of ORF57 crystals obtained in our experiments were used to collect X-ray diffraction data separately (S1 Fig). The spindle-shaped crystal crystallized in the reservoir contains 0.8 M Li_2_SO_4_, 0.1 M BIS-TRIS propane and other crystallized conditions shown in Table 1. The cubic crystal crystallized at very high concentrations of salt buffer contains 0.01 M MgCl_2_, 0.05 M HEPES (pH 7.0), 4.0 M LiCl. After data calculation, the cubic crystal consists only of monomers in the crystal asymmetric unit, while in the spindle shape crystal ORF57-CTD forms a dimer in the crystal asymmetric unit and the resolution was relatively lower (Table 2). As the cubic crystal crystallized in the buffer which has a very high concentration of the salt, it is possible that the dimer was dissolved into monomers by hydrophobic interactions during crystallization.

**Table 1:** The conditions for crystallization.

No.	Shape	Seeds	Buffer	Salt
1	Spindle	No	Phosphate pH 6.9	1.0 M NaH_2_PO_4_, 1.0M K_2_HPO_4_
2	Spindle	No	0.1 M BIS-TRIS propane pH 7.0	0.7 M (NH_4_)_2_C_4_H_4_O_6_
3	Spindle	No	0.1 M BIS-TRIS propane pH 7.0	0.6 M NaKC_4_H_4_O_6_
4	Spindle	No	0.1 M BIS-TRIS propane pH 7.0	0.8 M Li_2_SO_4_
5	Spindle	No	0.1 M Tris pH 8.5	0.8 M Li_2_SO_4_
6	Spindle	No	0.1 M Tris pH 8.5	1.8 M MgSO_4_
7	Spindle	No	0.1 M Tris pH 8.5	0.7 M (NH_4_)_2_C_4_H_4_O_6_
8	Cube	Yes	0.05M Na-HEPES pH 7.0	0.01 M MgCl_2_, 4.0 M LiCl

**Table 2:** Data collection and refinement statistics.

Data collection	Monomer	Dimer
Wavelength (Å)	1.2724	0.9792
Space group	I432	P6_4_22
Unit-cell parameters	a = b = c = 175.78Å	a = b = 167.53 c = 227.63Å
Resolution (Å)	3.06 (3.11–3.06)^a^	3.50(3.56–3.50)
unique reflections	9118 (440)	24306(1185)
Completeness (%)	100 (100)	99.8(99.9)
Redundancy	82.1 (85.8)	10.3(10.5)
Mean *I/ơ* (I)	112.3 (21.8)	26.4(3.7)
Molecules in asymmetric unit	1	2
*R*merge (%)	10.9 (52.5)	14.1(80.5)
*Structure refinement*		
Resolution range (Å)	47.0–3.06	33.2–3.51
*R*work/*R*free (%)	22.5/26.9	25.9/30.6
Average B factor (Å^2^)		
Main chain	49.62	113.9
Side chain	52.44	122.5
Number of atoms		
Residues	256	560
Protein	2018	4412
Ramachandran plot (%)		
Most favored	89.7	89.2
Allowed	8.4	9.9
Disallowed	1.9	0.9
R.m.s. deviations		
Bond lengths (Å)	0.012	0.004
Bond angles (°)	1.85	0.87

^a^ the values in parenthesis are for the highest resolution shell.

### Overall structure of ORF57-CTD

**Fig 1:**
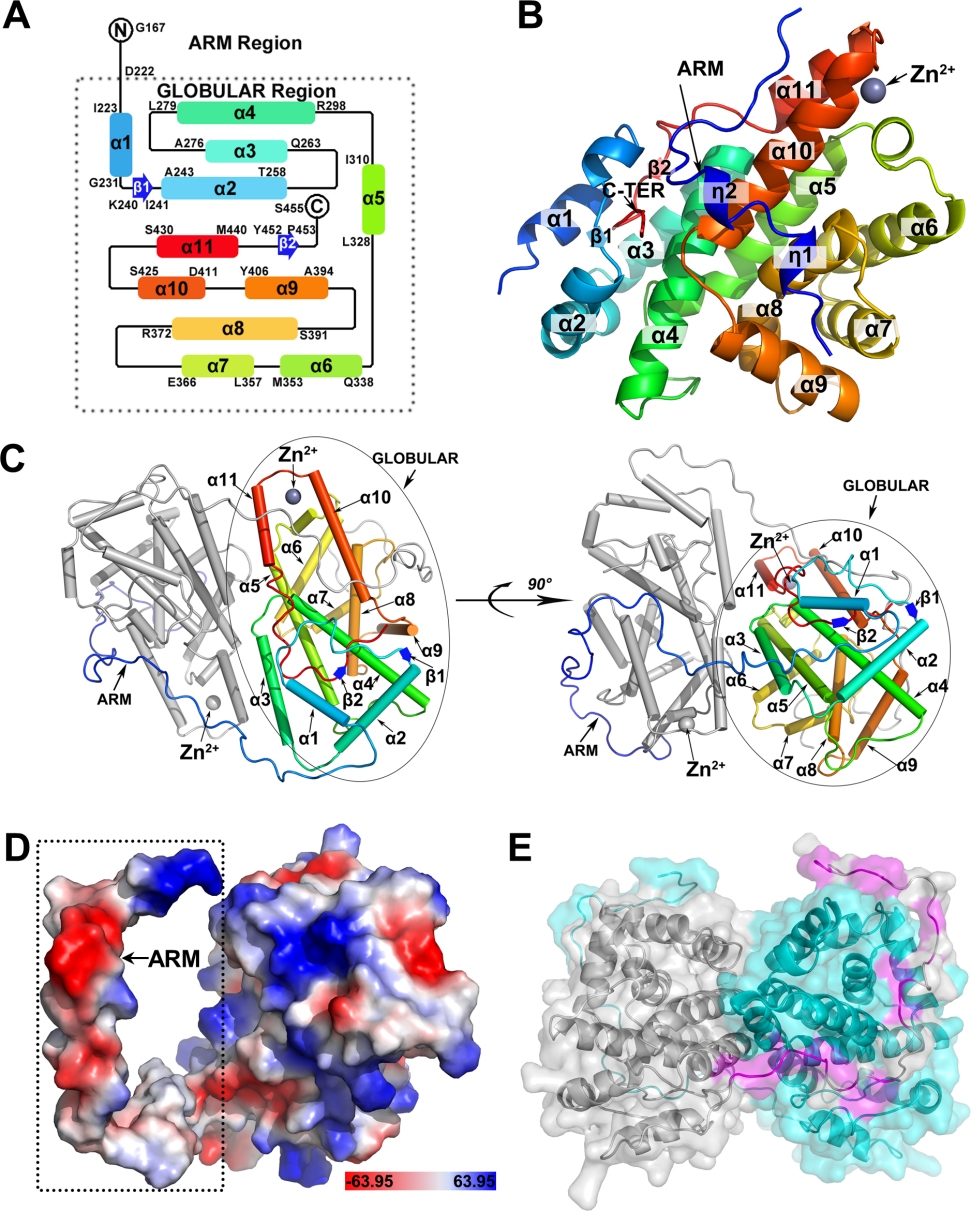
The crystal structure of KSHV ORF57-CTD in monomer and dimer. (A) Topology of the secondary structure of ORF57-CTD in a dimer form illustrates the residue ranges of the α-helix (rainbow blocks), β-sheet (blue arrows). (B) Monomeric structure of ORF57-CTD is shown in a cartoon model and colored with rainbow spectrum (α: α-helixes; β: β-sheet; η: η-helixes), the monomer structure harbors two additional η-helixes in the N terminus. Each monomer contains Zinc cation (Zn^2+^, grey sphere). (C) Dimeric structure of ORF57-CTD displays in two views rotated 90°. The helixes display as a cylinder, β-sheet display as an arrow. The monomer A and B are colored with rainbow spectrum and gray, respectively. Detailed in the colored monomer A are the numbered helixes within the globular domain and the “arm” region. (D) Electrostatic charges of the monomeric structure of dimerized ORF57-CTD (scaled color in PyMol). Red, negative charge; blue, positive charge. (E) The dimeric interface surface between the “arm” region and the globular domain displays in detail. The monomer A and monomer B are colored by grey and cyan, respectively. The hydrophobic residues of the left monomer of the “arm” region forming hydrophobic interaction are colored by magenta.

The observed monomer structure revealed that ORF57-CTD consists of 11 α-helixes and two short β-sheets (Fig 1A). The monomeric crystal belongs to space group I432 and the final structure (Fig 1B and S1 Movie) was refined at resolution of 3.06 Å with *R* and *R*free values to 22.5% and 26.9%, respectively (Table 2). As shown in Fig 1B, the structure of monomeric ORF57-CTD forms a globular domain which consisting of bundle of 11 α-helixes connected with unstructured loops of various sizes. The N-terminus is represented by an extended “arm” region lacking any α-helixes, but only two unstable small η-helixes (3_10_-helixes). The dimeric crystal belongs to space group P6_4_22 and the final structure (Fig 1C and S2 Movie) was refined at resolution of 3.5 Å with *R* and *R*free values to 25.9% and 30.6%, respectively (Table 2). The electron densities for residues V168-R189 and P211-D220 and S455 are not visible in the monomeric structure. Moreover, in the dimeric structure, only residues V168-L175 and S455 are unidentified because of the poor densities. The two monomers in the dimeric structure are nearly identical, and the root-mean-square deviation (RMSD) of atomic positions is only 0.528 Å when superimposed, but the N-terminus of the monomeric ORF57-CTD in dimer extends out to a different direction from the N-terminus of monomer if superimposed (S2 Fig). The dimeric structure is formed by the extended N-terminal “arm” region contacting with the globular domain of the neighboring monomer and shaping as a coffee bean conformation with approximate dimensions of 69.0 × 50.9 × 50.0 Å in the asymmetric unit, with a deep groove extends around the interface of the two globular domains. Each monomer contains a zinc ion chelated by four residues, the cysteines at the position 333, 427, 432, and the histidine at 423, which form a consensus zinc-binding motif. The N-terminal “arm” region of one monomer protrudes out on the globular domain of another anti-parallel monomer, while the C-terminal end is enclosed into the globular domain of the same monomer.

### The “arm” region of ORF57-CTD docks on the surface of the globular domain *in trans*

The N-terminal arm of ORF57-CTD (residues from 167–222) contains a number of highly hydrophobic residues (especially residues I, V and F) (Fig 1D and 1E and S3A Fig). The arm extends from the globular domain of the same monomer to dock in a canyon on the surface of the globular domain of the neighboring monomer like a barb (Fig 1C and Fig 1D). Thus, the arm region in the dimeric structure lacks two unstable small η-helixes. PDBePISA analysis by comparing with other interactions at the dimer interface showed that the “arm” region makes major contributions, mainly (>75%) by its hydrophobic residues, suggesting the “arm” region very likely participating the dimerization (S5 Fig).

### The C-terminus of ORF57-CTD inserts into the globular domain *in cis*

**Fig 2:**
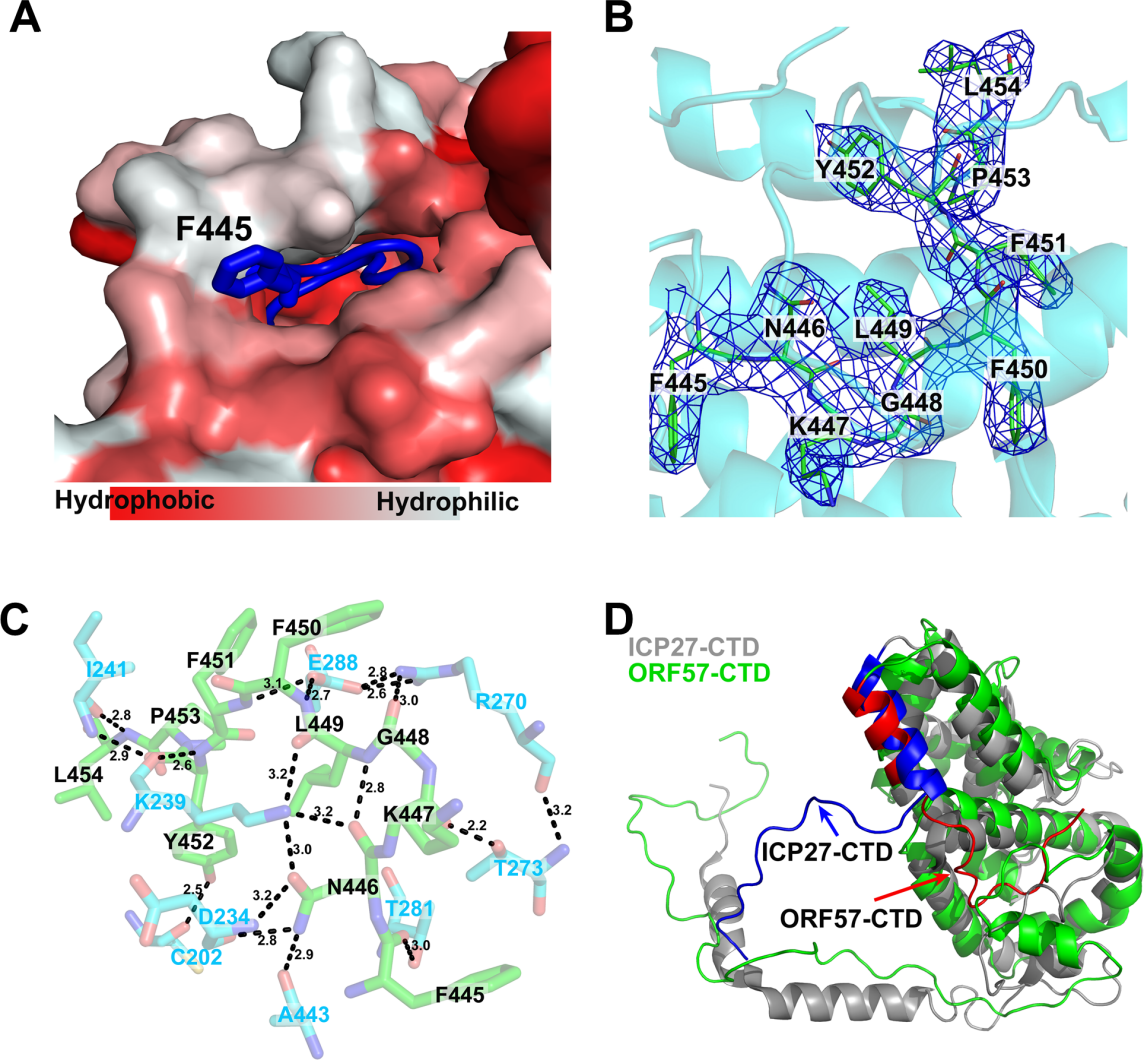
The C-terminal end structure of ORF57 and its difference from HSV-1 ICP27. (A) The C-terminal end F445-L454 inserts into the globular domain of the same monomer in cis (scaled color in PyMol with the ConSurf Server). (B) The electron density of the C-terminal end (green) provides the unambiguous conformation. (C) The interactions between the surrounding residues (light blue) and the C-terminal end (green) are shown in black dash lines with the distance (in Å). (D) The monomeric structure in dimerized ORF57 and ICP27-CTDs (PDB ID: 4yxp). The monomeric structures of ORF57-CTD (green) and ICP27-CTD (grey) as occur in a dimer are superimposed with the C-terminal ends of ORF57-CTD in red and ICP27-CTD in blue.

The C-terminus of ORF57-CTD (residues from 445 to 454) has a medium degree of conservation among herpesvirus homologues (S4 Fig). In the structure of ORF57-CTD, the C-terminal ends inserts into the same monomer (Fig 2A). The C-terminus is rich in hydrophobic residues (Fig 2A and S3B Fig) and forms a series of hydrogen bonds and hydrophobic contact to the surrounding residues (Fig 2B and 2C). The hydrophobic interactions between the C-terminus and the surrounding residues mainly come from the nitrogen and oxygen atoms of the backbones (Fig 2B and 2C). Specifically, the nitrogen atom of F451 in the backbone forms a hydrogen bond with OE2 atom of E288 and Y452 is stabilized by the residues K239, I241 and C202. The amino group of F450 also forms a hydrogen bond with OE2 of E288 (Fig 2C). F451 is highly conserved among all homologues (S4 and S8 Figs), although F450 is not as conserved as F451 (S4 and S8 Figs). The residue at the same position is usually an aromatic residue, such as phenylalanine and tyrosine, suggesting that selective pressures exist at this site (S8 Fig). L449, a less conserved residue (Fig 2C and S8 Fig), forms two hydrogen bonds through the nitrogen atom to the oxygen atom of N446 and the oxygen atom to the amine group of K239 side chain. G448, which is also rather conserved (Fig 2C and S8 Fig), forms a hydrogen bond between the oxygen atom and the NH1 group of R270. F445, N446 and K447 are not conserved (Fig 2C and S8 Fig). The carbonyl oxygen atoms of F445 and K447 interact with the OG1 atom of T281 and T273, respectively. As a single residue within the C-terminus, N446 provides most interactions. The ND2 atom of N446 contacts with the oxygen atoms of A443 and D234, the OD1 atom forms a hydrogen bond with the NZ atom of K239, and the oxygen atom is stabilized by K239 and L449 as described above. These interactions lock the C-terminus into the globular domain *in cis*.

### Comparison with the structure of HSV1 ICP27

DALI search indicates two similar structures (PDB ID: 4YXP and 5BQK) with our structures of ORF57-CTD. In fact, both are the structures of HSV1 ICP27-CTD, which determined by two research groups separately [21,22]. Although ORF57-CTD and ICP27-CTD have low protein sequence identities (~ 15%), their structures are quite similar (Fig 2D), with RMSD value of 2.8 Å for 174 superimposed Cα atoms, suggesting that their conserved roles in self-interaction possibly rely on their structures. However, there are two notable differences. First, in contrast to ICP27 “arm” domain containing 2 helixes, the ORF57 arm domain lacks such a notable α-helix in its “arm”. Second, the C-terminal residues 500–512 (YVHGKYFYCNSLF) of ICP27-CTD docks on the neighboring monomer *in trans* and is required for dimerization [21,22]. In contrast, the C-terminal residues 445–454 (NKGLFFYPL) of ORF57-CTD is locked in the same monomer *in cis* and thereby is not directly involved in dimerization.

### The structural determinants of ORF57 dimer formation

**Fig 3:**
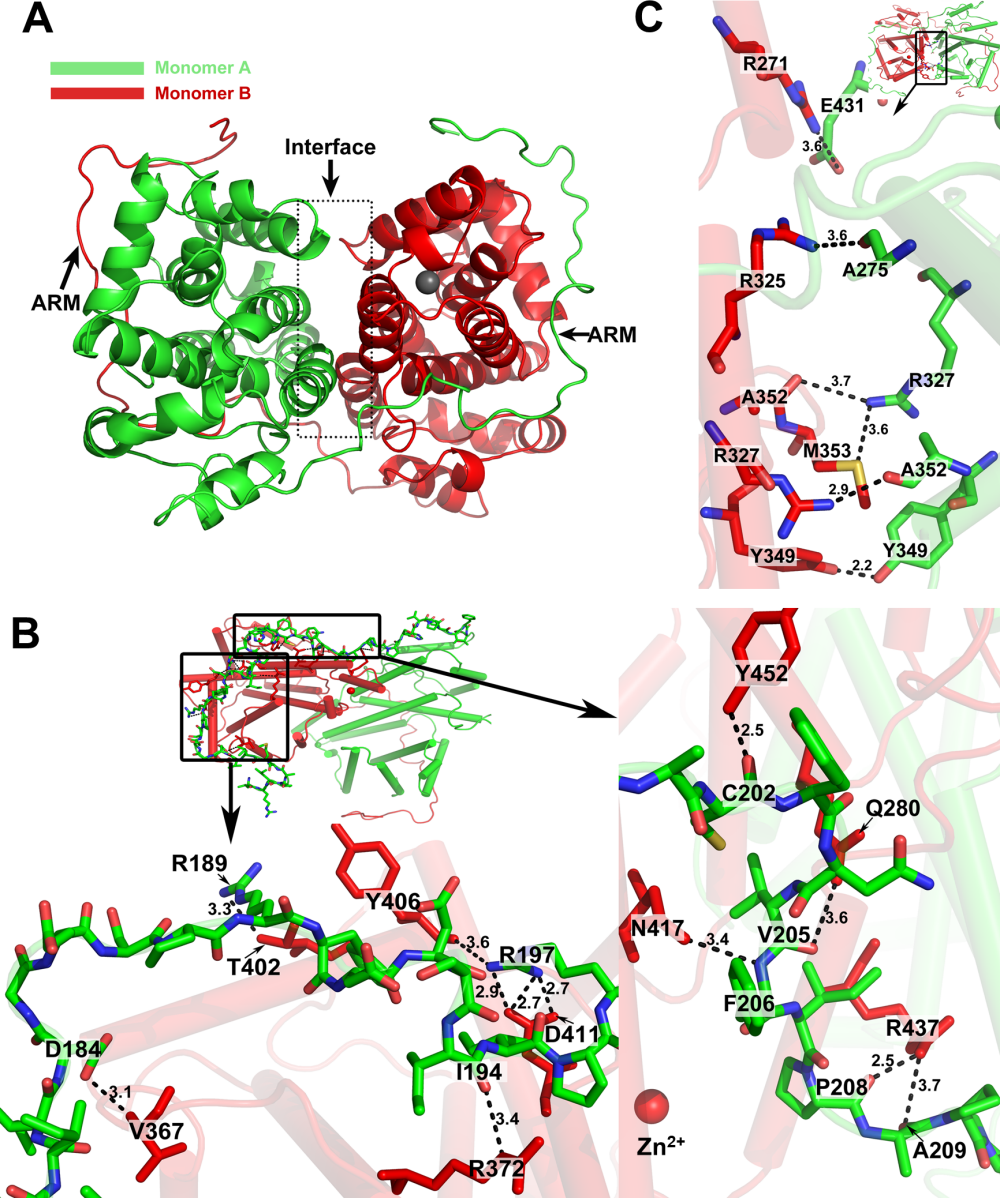
The structure determinants in ORF57 dimer formation. (A) A 3D diagram of ORF57-CTD in a dimer. Two monomers are depicted with green and red respectively. Arrows point to the two distinct interaction surfaces: (1) the interaction between the “arm” region and the outer surface of the other monomer and (2) the mutual interaction of two globular domains at the interface (dashed box). (B and C) The hydrogen bonds between two monomers contribute to dimerization. The interaction at the interface between the “arm” region (green) and the globular domain (red) is mediated by a large number of residues within the “arm” region and the surface of the globular domain (B).The interaction at the interface between the globular domains (colored in green and red respectively) is limited only to several residues (C). The distances between individual residues are showed in Å.

**Fig 4:**
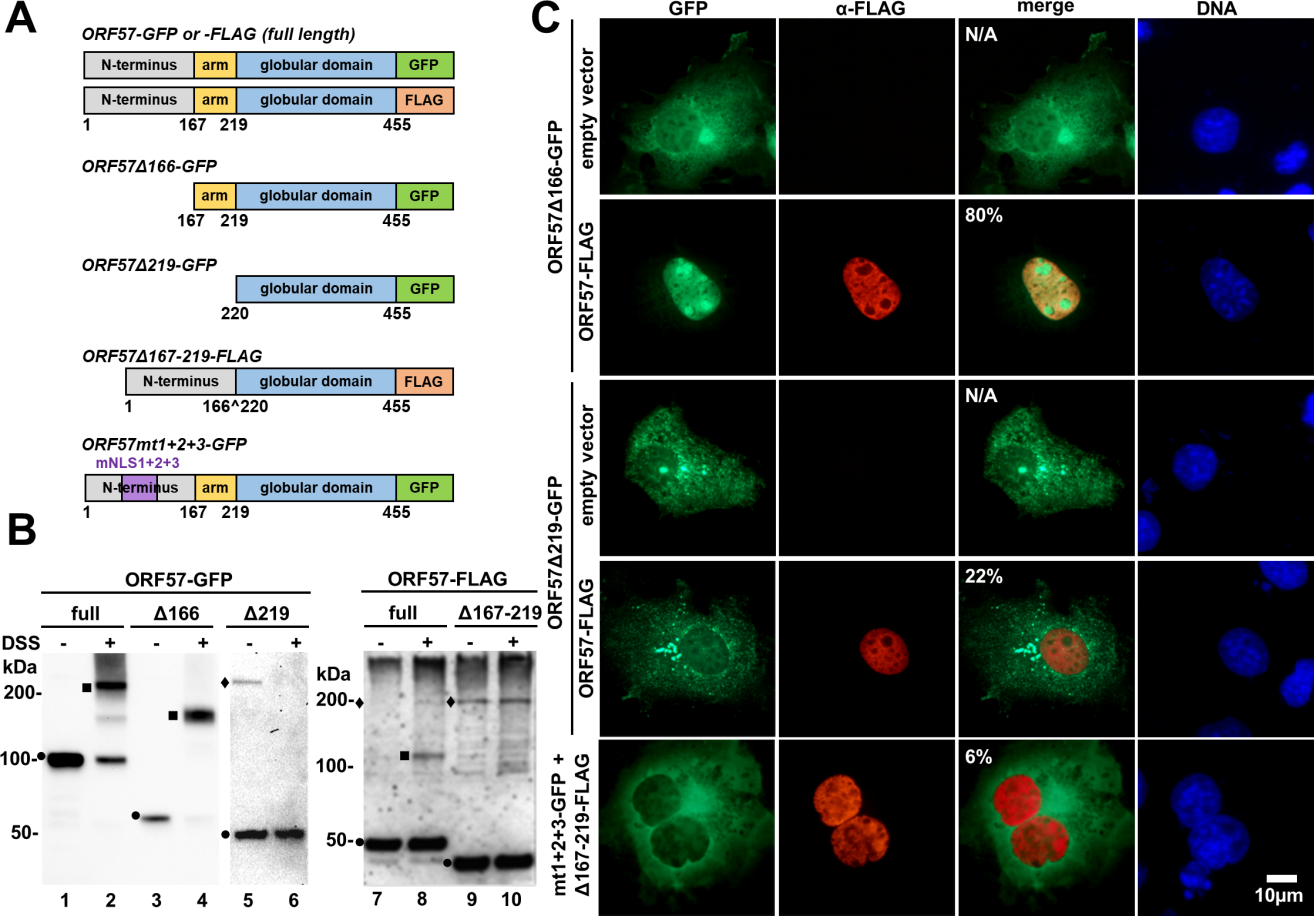
Deletion of the “arm” region abrogates ORF57 dimerization. (A) The constructs of GFP-tagged ORF57, FLAG-tagged ORF57 and its truncation mutants used in the study. (B) In vitro crosslinking assay of full-length or mutant ORF57. HEK293 cells transfected with indicated ORF57 expression vectors were harvested at 24 h post transfection and the cell resuspended in PBS were treated with 50 μM in final concentration of DSS protein crosslinker for 30 min, with DMSO vehicle (-) as a control. The dimer formation was monitored by Western blotting using anti-GFP (lanes 1–6) or anti-FLAG (lanes 7–10) antibodies. Circular symbols, monomer; square symbols, dimer; diamond symbol, the nonspecific protein band. (C) COS-1 cells seeded on glass cover slips were cotransfected with a GFP-tagged arm-null mutant (ORF57Δ219-GFP) and an empty vector or FLAG-tagged full-length ORF57 (ORF57-FLAG). The arm-bearing, dimerization-competent mutant (ORF57Δ166-GFP) was used as a positive control. The FLAG-tagged mutant with internal arm deletion (ORF57Δ167-219-FLAG) was cotransfected with a dimerization–competent mutant full-length ORF57-GFP containing three disrupted nuclear localization signals (ORF57mt1+2+3-GFP). Localization of GFP-tagged proteins (green) was determined using direct fluorescence microscopy. The FLAG-tagged proteins (red) were visualized by indirect immunofluorescence staining using anti-FLAG M2 antibody. Cell nuclei (blue) were counterstained with Hoechst 33342 dye. Indicated in the merged panel are % cells with nuclear translocation from 50 double-color positive cells. N/A, not applicable.

Based on these structural features, we concluded that each ORF57 monomer consists of two structurally distinct domains: (1) the “arm” region covering residues 167–219 and (2) the globular domain consisting of 11 helixes covering the rest of ORF57-CTD. The connection between the two monomers is mediated via multiple hydrophilic interactions including hydrogen bonds and salt bridges and form two major interaction surfaces (S5 Fig). One interface is the interaction between the “arm” region and the surfaces of the globular domain, and the other interface is the interaction between the two globular domains (Fig 3A). The arm-globular interface comprises a large number of residues and bonds (Fig 3B), whereas the globular-globular interface has fewer residues and bonds (Fig 3C). To determine the roles of each interface in ORF57 dimerization, we constructed an arm-null-mutant Δ219 expressing only the globular domain and an internal arm deletion mutant Δ167–219 in the context of full length ORF57 (Fig 4A) and demonstrated that the arm-null ORF57 is labile and deficient from dimerization (Fig 4B and 4C).

As shown in S6A Fig, the FLAG-tagged, arm-null Δ219 mutant displayed a little protein expression only in HEK293 cells treated with proteasome inhibitor MG132 when compared with wt ORF57 fusion (compare lanes 3–4 to 1–2), despite the similar RNA levels were seen in all three constructs (S6B Fig). This indicates that deletion of the arm from ORF57 leads to high instability of the arm-deletion mutant. To overcome this problem, we fused ORF57 with GFP after mutation of cryptic splice sites leading to alternative splicing of ORF57 GFP[29], this led to partial stabilization (S6C Fig) of ORF57 arm-null mutant. The capability of these proteins to form dimers in the presence or absence of DSS crosslinker was initially compared by Western blot analysis using cell lysates derived from the corresponding expression vector-transfected HEK293 cells. As expected, the full-length ORF57 and the arm-bearing Δ166 mutant efficiently formed homodimers as seen in the previous publication [18], but the arm-null Δ219 and Δ167–219 mutants failed to form a homodimer (Fig 4B, compare lanes 5–6 to 1–4 and lanes 9–10 to 7–8), suggesting the important role of the “arm” region in ORF57 dimerization.

The arm-null Δ219 and Δ167–219 lacking of dimerization was further confirmed in COS-1 cells by nuclear translocation assays. As shown in Fig 4C by cotransfection of the GFP-tagged, arm-null Δ219 or arm-bearing Δ166 with Flag-tagged wt ORF57, we found the cytoplasmic arm-bearing Δ166, but not the cytoplasmic arm-null Δ219, could dimerize with wt ORF57 in the cytoplasm and subsequently was translocated into the nuclear compartment (80% vs 22%, Fig 4C). This observation was strongly supported in a separate experiment by using a mutant ORF57 with an internal arm deletion of which the mutant remains as a nuclear protein. In the following nuclear translocation assays, the mutant with internal arm deletion also showed no dimerization with a GFP-tagged, cytoplasmic mutant ORF57 mt1+2+3 which contains the arm and an intact C-terminal domain (Fig 4C). We saw only 6% of double positive cells displaying some degrees of nuclear translocation. Altogether, our data indicate that lack of the arm region prohibits heterodimerization of ORF57-GFP from ORF57-FLAG.

### Contributions of the interface residues to ORF57 dimerization

**Fig 5:**
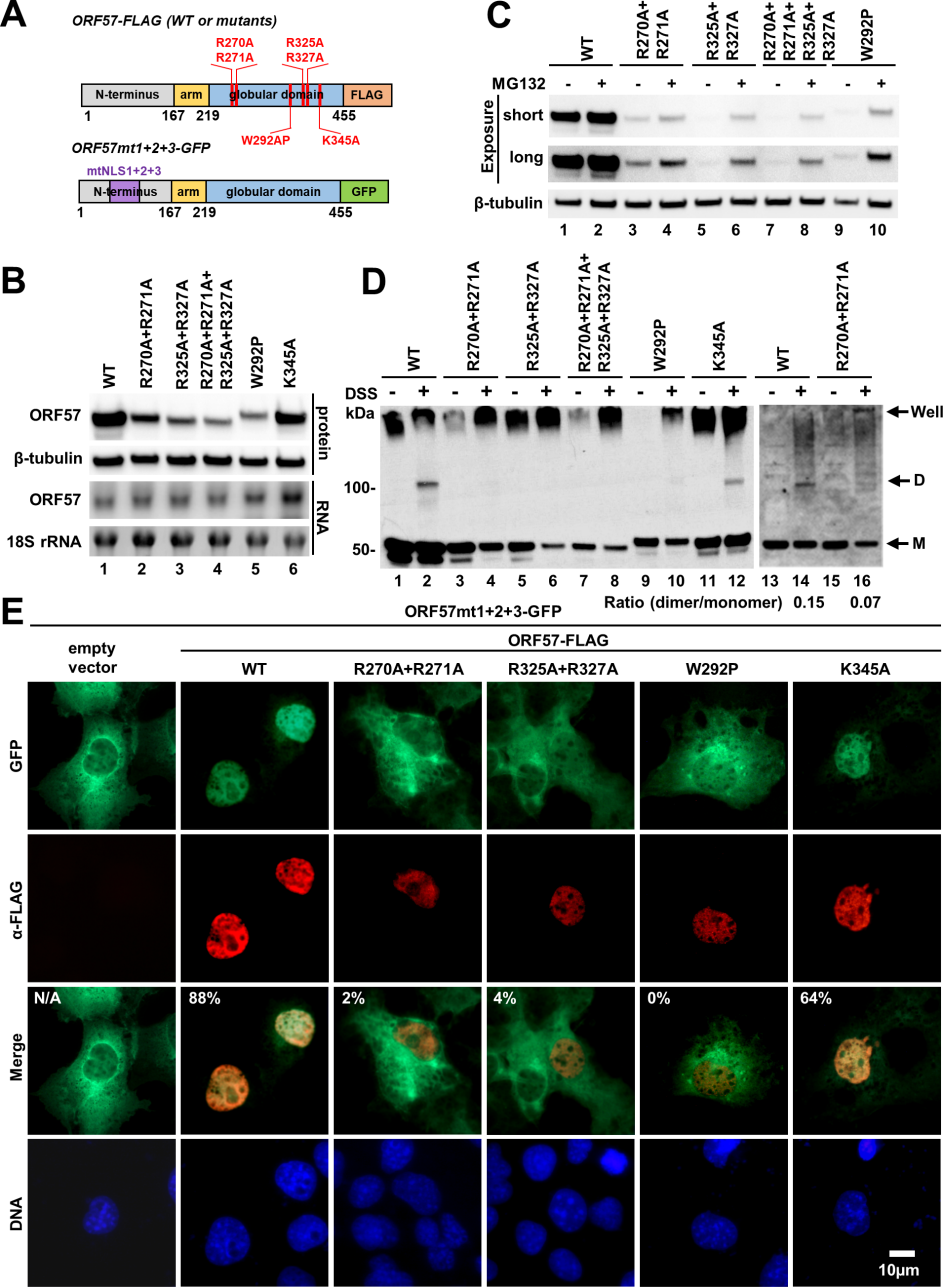
Point mutation of the globular-globular interface residues impairs ORF57 dimerization. (A) Representative diagrams of the constructs encoding C-terminal FLAG- or GFP-tagged ORF57 and its mutants. (B) Effect of interface point mutations on ORF57 expression. HEK293 cells transfected with indicated FLAG-tagged ORF57 expression vectors were harvested at 24 h after transfection for extraction of total proteins and RNA. The protein levels of ORF57 or its mutants were determined by Western blotting using anti-FLAG antibody (top). Cellular β-tubulin was used as a loading control. The RNA levels were determined by Northern blotting (bottom). The level of 18S rRNA was used to ensure equal RNA loading. (C) ORF57 interface mutants are partially degraded through proteasome pathway. HEK293 cells were transfected with indicated ORF57 expression vectors and treated at 20 h after transfection with MG132 for additional 4 h or with DMSO (vehicle) as a control. The cell lysates were examined for ORF57 protein by Western blotting using anti-FLAG antibody. Cellular β-tubulin was used as loading control. (D) ORF57 mutants with point mutation of the globular-globular interface residues are incapable of protein dimerization. HEK293 cells were transfected with equal amount (2 μg) of the vectors expressing either wild type (WT) or globular domain mutant ORF57-FLAG. The cells at 24 h after transfection were treated with proteasome inhibitor MG132 for 6 h (lanes 1–12). Alternatively, the expression of wild type and mutant protein was adjusted by transfection of variable amount of the plasmid DNA with 0.5 μg for WT and 2 μg for mutant (lanes 13–16). The cells were harvested in PBS and exposed to chemical crosslinker DSS (50 μM in final concentration) or DMSO (vehicle) for 30 min. The dimer formation was monitored by Western blotting using an anti-FLAG antibody. M, monomer; D, dimer. (E) COS-1 cells seeded on glass cover slips were cotransfected with a GFP-tagged ORF57 mt1+2+3 (a three nuclear localization signal-deficient mutant, mt1+2+3) and a FLAG-tagged full-length of ORF57 interface mutant. The empty vector was used as a negative control and ORF57 WT as a positive control. The previously reported dimerization-incompetent ORF57-W292P and dimerization-competent ORF57-K345A were also used as controls. The localization of GFP-tagged proteins (green) was detected using direct fluorescent microscopy. The FLAG-tagged proteins (red) were visualized by indirect immunofluorescent staining using an anti-FLAG M2 antibody. Cell nuclei (blue) were counterstained with Hoechst 33342 dye. Indicated in the merged panel are % cells with nuclear translocation from 50 double-color positive cells. N/A, not applicable.

The globular domain of ORF57-CTD consists of 11 helixes and forms the contact interface in the center of the dimer (Fig 3A and 3C). Globular-globular interaction is mediated via a few hydrogen bonds between several residues (Fig 3C and S5 Fig). To determine their contributions to dimerization, we constructed a set of point mutations in the context of full-length ORF57 (Fig 5A). Specifically, we mutated R271 to A271, involved in interaction with E431. The neighboring R270 was also simultaneously mutated to A270 to avoid possible charge compensation after R271 mutation. In addition, we simultaneously mutated two neighboring arginines (R325 and R327) to alanine to prevent R325 from interacting with A275 and R327 from interacting with A352 and M353 (Fig 3C and S5 Fig). In these studies, we used a dimerization-incompetent W292P mutant and a dimerization-competent K345A mutant as controls [18]. All mutants were cloned and expressed as a C-terminal FLAG-tagged fusion protein (Fig 5A). The equal amount of the wild-type or mutant ORF57 expression vector was transfected into HEK293 cells separately. The level of expressed ORF57 proteins and corresponding mRNA was determined by Western and Northern blotting, respectively. As shown in Fig 5B, all ORF57 with interface mutations, similar to the dimerization-incompetent mutant W292P, displayed significant reduction in the protein levels in comparison to the wild-type ORF57 and the dimer-competent K345A, although no obvious changes were observed in their RNA levels by Northern Blotting. This indicates that each interface mutant was transcribed efficiently, but the mutant protein was unstable. To test whether the mutant protein was degraded via a proteasome-mediated pathway, we next compared their expression in the presence or absence of a proteasome inhibitor MG132 (Fig 5C) and showed a partial restoration of the expression, similarly to the W292P mutant. To further examine whether the protein instability of each interface mutant was caused by loss of its dimerization activity as seen for the W292P mutant [18], we performed the *in vitro* crosslinking assay after the expression of each mutant in HEK293 cells. To eliminate the possibility that the lack of dimer detection was because of low level of mutant protein expression due to its instability, we performed the same assay with the protein expressed in HEK293 cells in the presence of MG132 and confirmed lack of dimerization of all interface mutants, as seen for the W292P, was not because of their protein expression level after carefully normalizing the expressed protein amount from individual expression vectors (Fig 5D, compare lanes 3–10 and 15–16 to lanes 1–2, 11–12 and 13–14).

To further verify the inability of ORF57 interface mutants to form dimer, we performed the nuclear translocation assay in COS-1 cells by cotransfection of the individual interface mutants along with a GFP-tagged full-length ORF57 with all three disrupted nuclear localization signals (NLS) (ORF57 mt1+2+3) [7] (Fig 5A). Mutation of all three NLSs impairs ORF57 nuclear localization and accumulates the GFP-tagged protein as a dysfunctional, but dimerable protein in the cytoplasm. Thus, the GFP-tagged ORF57 mt 1+2+3 could be translocated into the nucleus upon coexpression with wild-type ORF57 containing functional NLSs through dimerization, with 88% efficiency [7]. Although both R270A/R271A and R325A/R327A were nuclear proteins, similarly to the dimerization-incompetent mutant W292P [18], they were unable to form a dimer with the ORF57 mt1+2+3 and thus were incapable (only 2% and 4% efficiency) to translocate the GFP-tagged cytoplasmic ORF57 mt1+2+3 into the nucleus when expressed. In contrast, the dimer competent K345A retained its ability to translocate the GFP-tagged mutant into the nucleus (66% efficiency) by dimerization, close to what we seen for the wild-type ORF57 (Fig 5E and S7 Fig).

In conclusion, the interface residues R270A/R271A and/or R325A/R327A in the CTD globular domain are essential for ORF57 dimerization. The mutation of these residues prevents dimer formation and consequently induces protein instability. However, the mutation of K345A in the vicinity of R325, R327 and Y349 residues (Fig 3C and S11 Fig) had no effect on ORF57 stability, dimer formation, and functionality, indicating that this residue is non-essential for ORF57 dimer formation via the C-terminal globular domain.

### The zinc-binding motif is important for protein stability of ORF57 and its homologues

**Fig 6:**
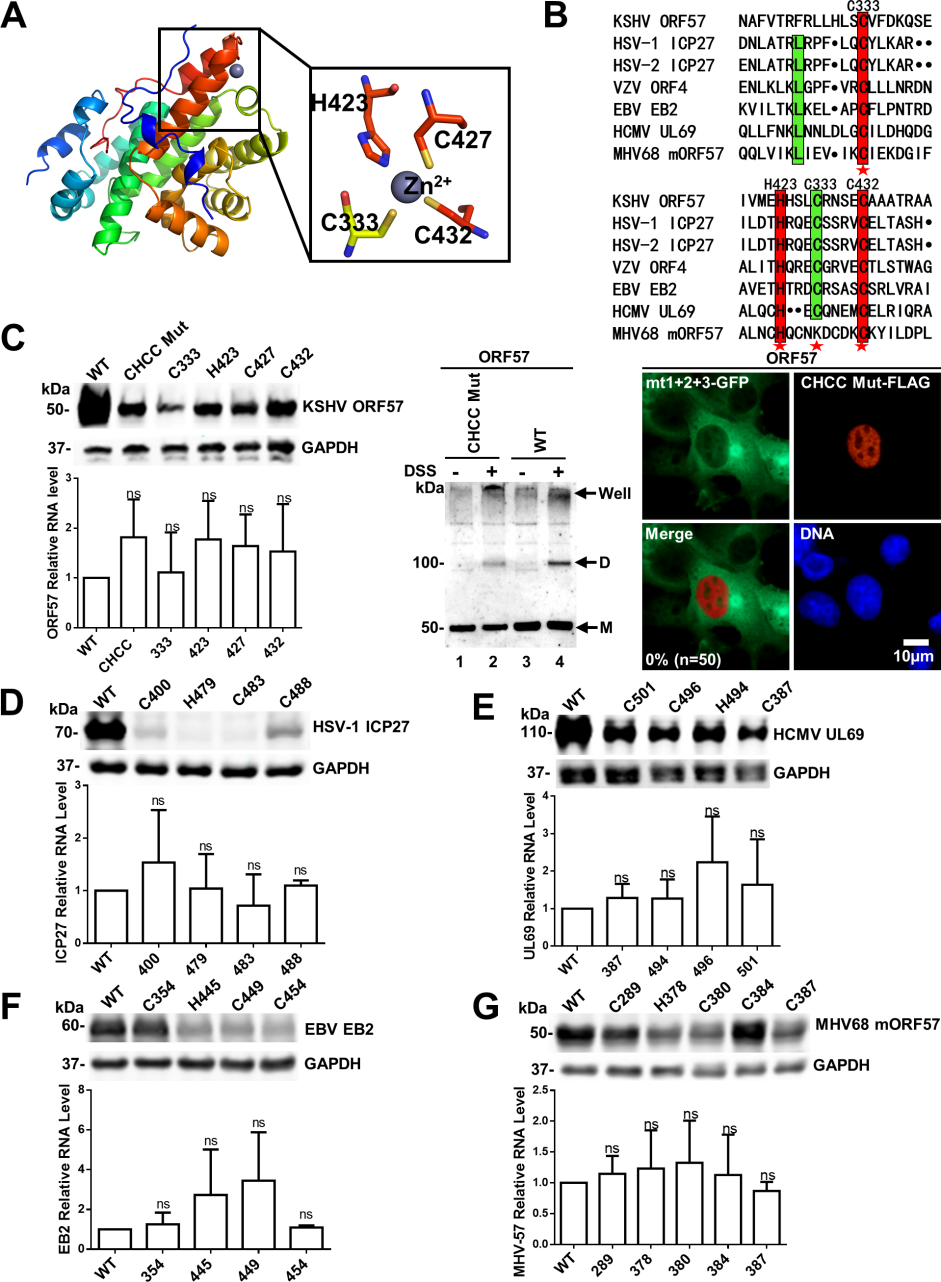
The zinc-binding motif is important for accumulation of KSHV ORF57 protein and its herpesvirus homologues. (A) The structure of ORF57-CTD in a cartoon model with rainbow spectrum. The zinc (Zn^2+^) ion (grey sphere) and the surrounding cysteine (C333, C427, C432) and histidine residues (H423) are highlighted and displayed as sticks.(B) Multiple alignment of the protein sequences was performed by Clustal Omega, including the C-terminal domain of KSHV-ORF57, ICP27 (herpes simplex virus type 1 and type 2, HSV1-ICP27 and HSV2-ICP27), ORF4 (varicella-zoster virus, VZV-ORF4), EB2 (Epstein-Barr virus, EBV-EB2), UL69 (human cytomegalovirus, HCMV-UL69), and mORF57 (murine gamma herpesvirus 68, MHV68-mORF57). Conserved residues are labeled in red, identical residues are highlighted in green, and the residues of the zinc-binding motif are marked by red stars. (C) Disruption of the zinc-binding motif reduces the stability and dimerizationin of KSHV ORF57 protein. HEK293 cells were transfected with KSHV ORF57 expression vectors with indicated serine for cysteine and leucine for histidine substitutions in the zinc-binding motif and harvested at 40 h post transfection. The cells were separated into two parts: one for Western blotting (top left panel) and one for RNA analysis (lower left bar graph). WT, wild type; CHCC Mut, a combined serine and leucine substitutions of all four residues in the zinc-binding motif. ORF57 with combined mutations of all four zinc-binding residues was tested for its dimerization activities both in vitro crosslinking and nuclear translocation assays (right two panels) as described in Fig 5D and 5E. M, monomer; D, dimer. (D-G) The zinc-binding motifs in other herpesvirus homologues are important for their protein stability. The similar serine and leucine substitution experiments were conducted to analyze the protein stability of HSV-1 ICP27 (D), HCMV UL69 (E), EBV EB2 (F) and MHV68-mORF57 (G). Results (mean ± SD) are representative of three independent experiments. NS, not significant.

A zinc-binding motif is a relative small structural motif and is characterized by the coordination of one or more zinc ions to stabilize protein [30,31]. As shown in Fig 6A, the zinc-binding motif coordinated by residues C333, H423, C427 and C432 was identified within the ORF57-CTD, with H423, C427 and C432 being located in a compact helix-loop-helix motif (from helix 10 to 11). Specifically, H423 and C432 are positioned on the helix 10 and 11, respectively, while C427 is resided on the interconnecting loop. The forth residue C333 within the loop linking helix 5 and 6, is positioned approximate 100 residues upstream to the other three (H423, C427 and C432) in the sequence. The zinc ion is chelated in the tetrahedral geometry, in which the bond length for Zn-S ranges from 2.2 to 2.4 Å and for Zn-N is about 2.1 Å. The alignment analysis indicated that this CHCC motif is highly conserved among ORF57 homologues from different herpesviruses (Fig 6B and S8 Fig). Due to its location buried inside the globular domain, we hypothesized that this motif may contribute to the stability and correct protein folding. To test this hypothesis, we mutated cysteines at position 333, 427 and 432 to a serine, and histidine at the position 423 to a leucine separately or in combination in the context of full-length ORF57. The expression plasmids were transiently transfected into HEK293 cells and then the expression levels of the mutants were determined by Western blotting for ORF57 protein and real-time PCR for ORF57 RNA. The wild-type ORF57 protein exhibited a much higher protein level compared to its mutants (Fig 6C, top left panel), though no significant differences were observed on their transcriptional level (Fig 6C, lower left bar graph). To examine whether CHCC motif mutant had a short half-life, we compared the CHCC mutant with the wt ORF57 in HEK293 cells in the presence of cycloheximide (CHX) for indicated time (S9 Fig), the Western blot results revealed that the CHCC mutant protein display a shorter half-life over the wt ORF57.

We further investigated if the zinc- binding motif is involved in ORF57 dimerization, we used the same methods described in Fig 4 and Fig 5 and revealed that the CHCC mutant can be weakly dimerized in chemical cross-linking assays, but lacks the nuclear translocation activity for the cytoplasmic mutant ORF57mt1+2+3 (Fig 6C, right panel). Altogether, these data suggest an important role of the CHCC motif in ORF57-CTD folding and stability.

Since the zinc-binding motif is highly conserved among ORF57 homologues (Fig 6B and S8 Fig), we further investigated possible roles of the zinc-binding motifs in the protein stability of the homologues from other herpesviruses. By conversion of the cysteine to serine and the leucine to histidine in the four residues of the zinc-binding motif within ICP27 (herpes simplex virus type 1), EB2 (Epstein-Barr virus, EBV), UL69 (human cytomegalovis, HCMV) and mORF57 (murine herpesvirus 68, MHV68) as described for KSHV ORF57 (Fig 6B and S8 Fig), we demonstrated that the predicted zinc-binding motifs in ICP27, EB2, UL69 and mORF57 are all important for high levels of the protein expression, but no significant contribution to transcription (Fig 6D–6G). These data provide the compelling evidence that the four residues in the zinc-binding motif of each homologue are important for their protein stability.

### The zinc-binding motif of ORF57 is important for viral gene expression during lytic replication

**Fig 7:**
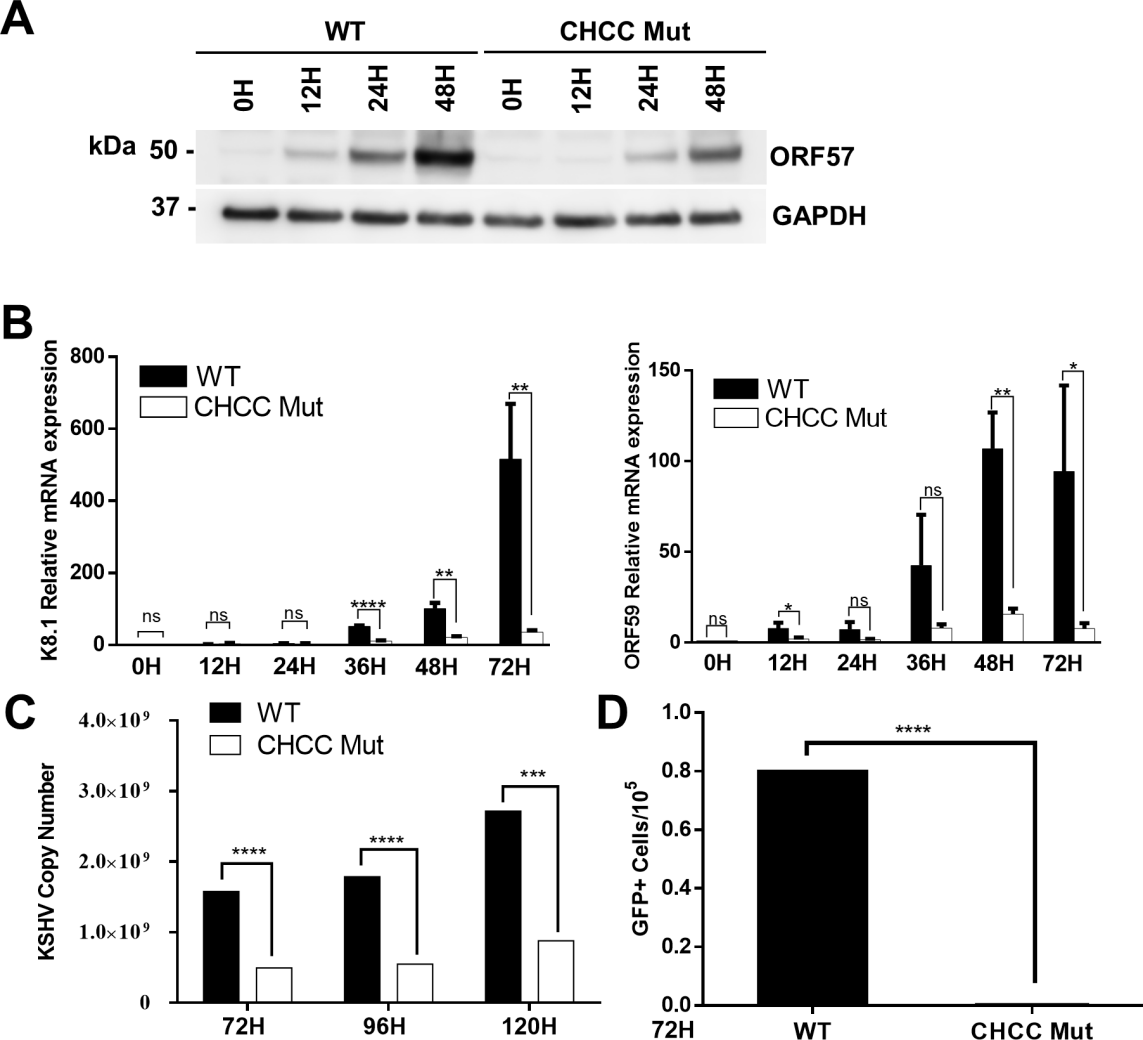
Mutation of the zinc-binding motif affects viral gene expression and KSHV production. WT and zinc-binding motif (CHCC Mut) mutated BAC16 were transfected into iSLK-puro cells, and the Hygromycin-resistant cells were induced with DOX and harvested at the indicated time (H). The ORF57 protein level from each BAC16 was determined by Western blotting using anti-ORF57 antibody. The endogenous GAPDH was used to ensure an equal loading (A). In parallel, the RNA levels of ORF59 and K8.1 were analyzed by RT-qPCR (B). (C-D) The CHCC mutant virus exhibits poor replication and virus production. The iSLK culture supernatant after lytic induction for 72, 96 and 120 h was quantified by qPCR for KSHV copy numbers. (D) FACS analysis of GFP-positive HEK293T cells after inoculation with concentrated viruses. WT and CHCC mutant virus were induced for 72 h and incubated with HEK293T cells for 24 h. Results (mean ± SD) are representative of three independent experiments, *p < 0.05, **p < 0.005, ***p < 0.0005, ****p < 0.00005. NS, not significant.

Given that the zinc-binding motifs is highly conserved and has similar function for protein stability among different herpesviruses, it is possible that this motif is critical for the function of ORF57 stabilizing viral mRNA and promoting viral gene expression during KSHV lytic replication. To test this hypothesis, we substituted the four residues within the zinc-binding motif of ORF57 as described in Fig 6C in the context of BAC16 and constructed the mutant genome of the KSHV recombinant virus [32] (S10 Fig). The integrity of BAC16 was analyzed by digestion with *Xho*I shown in S10 Fig and no additional mutations were found when compared with the wild-type genome. The wild-type and recombined BAC16 clones were transfected into iSLK cells and screened by Hygromycin B. The resistant stable cell lines were exposed to doxycycline (DOX) to reactivate KSHV lytic replication, and the cells were harvested at the indicated time points for analysis. When compared with wild-type KSHV virus, the mutant virus expressed much less amount of ORF57 protein during lytic induction (Fig 7A). Remarkably, the mutant virus also exhibited severe reduction of K8.1 and ORF59 RNAs, two major targets of ORF57 [9] (Fig 7B), accompanied by significant reduction of KSHV DNA replication and virus production (Fig 7C and 7D). The deficiency in expression of lytic gene products and viral replication caused by the zinc-binding motif mutation is quite similar to that caused by ORF57 deletion [9]. Taken together, these results demonstrated that the identified zinc-binding motif is critical for the expression and function of ORF57 to promote KSHV lytic replication.

## Discussion

In the present study and for the first time, we resolved the 3D structure of the KSHV ORF57-CTD. In our experimental system, direct expression of the full-length ORF57 in *Escherichia coli* led to extremely low yield. After optimization of the conditions, the N-terminal truncation mutant retaining the residues 167 to 455 (ORF57-CTD) became relatively stable and was successfully crystallized. Finally, we obtained a dimeric structure at 3.5 Å and a monomeric structure at 3.0 Å through X-ray crystallography.

ORF57-CTD monomer comprises 11 helixes which is generally in agreement with the predicted secondary structure by biochemical analyses [19]. The solved structure of ORF57 protein is quite different from the folding structure from I-TASSER database [33] by Taylor [34], in which the dimer is formed between the core domains. The distinguishing feature of ORF57-CTD is the “arm” region which extends to the neighboring monomer *in trans* and the C-terminal residues 445–454 (NKGLFFYPL) which insert into the globular *in cis*. The conformation of the “arm” region of ORF57 is similar to the N-terminal “arm” region of ICP27-CTD from HSV-1 [21,22]. However, the N-terminal “arm” region of ICP27-CTD forms two α-helixes, while the “arm” region of ORF57-CTD avoids any major structural motifs. Interestingly, the C-terminal end of ICP27-CTD, which also has an “arm” region extending to the neighboring monomer *in trans*, is essential for the dimerization, whereas the C-terminal end of ORF57-CTD inserts itself into the globular domain *in cis* to restrict itself from interacting with another monomer (Fig 2D), suggesting that the residues in the C-terminal end might not be commonly used for dimerization among all ORF57 homologues.

The self-association and homodimer formation are common features in eukaryotic proteins. Structural and biophysical studies show that dimer formation plays an important role in protein functions [35]. Homodimerization improves protein stability, regulates enzyme activities, and increases interaction complexity. In general, most dimerization is mediated by noncovalent interactions. Contact interaction and domain swapping are two major forms of noncovalent interactions. Contact interaction is mainly mediated by several α-helix in one monomer, which form stable contacts with the other monomer. In our case, the homodimerization of ORF57-CTD is mediated by contact interactions at both arm-globular and globular-globular interfaces.

One striking difference between this study and the mutagenesis data on ICP27 is that dimerization of KSHV ORF57 is more vulnerable to amino acid substitutions than ICP27 at the intermolecular interactions [22]. This difference could be attributable to the ICP27 dimerization stabilized by an extensive network of intermolecular contacts, while ORF57 dimerization is stabilized only by its arm and a few binding interface between the globular domains (S5 Fig). Moreover, the surface electrostatic potential of the "arm" region that mediates intermolecular interactions also differs in some degree between ORF57-CTD and ICP27-CTD. The "arm" region of ORF57-CTD is rich in negative charged residues as compared with ICP27, indicating the different binding stability for dimerization of two proteins (S12 Fig). We demonstrated that disruption of even one single hydrogen bond (R270A+R271A) at the globular interface is sufficient to abolish ORF57 dimerization (Fig 5). Alternatively, these mutations (R270A+R271A) may completely change the local structures, more than just disrupting a signal hydrogen bond. In contrast, introduction of single substitutions along the binding surface of ICP27 had no effect on dimer formation [22]. More interestingly, the vulnerable feature of KSHV ORF57 structure in dimer formation could be revealed by point mutations even in other regions (E287, E288, W292) not responsible for intermolecular contacts [18]. In the current structure, the E287, E288 and W292 residues locate inside α4 helix in the center of the globular domain and interact with residues 407, 410, 412, 270, 309 within the monomer, but they do not interact with any residue from another monomer (S11A Fig). Thus, the mutation of these residues may result in the whole structure collapse. That the highly conserved residue E288 interacts with the C terminal end residues (Fig 2C) presumably contributes to maintain ORF57-CTD structure. One exception is the K345A mutation in our previous study [18]. In this report, we found that K345 locates in α-helix 6 and the side chain of K345 protrudes out to the surface of the dimeric structure and not mediates interaction with the other monomer (S11B Fig).

Previous study reported that several small compounds with special design inhibit the activity of KSHV protease (KSHV Pr) through disrupting dimer formation [36–38]. KSHV Pr, which plays an important role in capsid maturation, is structural and functional conserved among all three human herpesvirus subfamilies [39–43]. Monomeric KSHV Pr is inactive and partially disordered, but KSHV Pr becomes active in dimeric state. As the C-terminal domain of KSHV Pr plays a key role in dimerization [44], the designed compounds bind to the dimer interface of KSHV Pr and disrupt dimer formation to fulfill the inhibition. HIV protease (HIV Pr) is also a homodimeric enzyme and essential for viral assembly during viral production, and the dimeric structure consists of a four-stranded antiparallel β-sheet from the N-terminus and C-terminus of each monomer[45]. Previous research designed a non-peptide-based linker to connect the N-terminus of each monomer, resulting in dimer dissociation and providing a potency strategy against HIV infection [46,47]. Like KSHV Pr and HIV Pr, ORF57 is also dimerized, and disrupting dimer formation results in swift protein degradation [18]. Therefore, our structure provides novel clues for compound development targeting the dimer interface of ORF57, finally preventing viral replication efficiency.

The zinc-binding motif CHCC is a classical protein folding motif found in some pathogenic viruses and eukaryotic cells [30,31,48,49]. The motif is mainly found in the nucleocapsid proteins of some retroviruses [50,51]. The nucleocapsid protein of HIV-1 and HIV-2 contains two CHCC motifs that are critical for packaging the viral RNA [52,53]. Glycoproteins of hantaviruses also forms two CHCC motifs [54], which are critical for viral replication and assembly. ORF57-CTD and ICP27-CTD harbor a zinc ion by this CHCC motif [21,22] which is highly conserved among herpesviruses. The zinc ion chelated by the histidine and cysteines modulates the stability of ORF57. Therefore modifying the zinc-binding motif among ORF57, EB2, UL69 and mORF57 would result in dramatic reduction in protein levels. In this report, we demonstrated that disruption of the CHCC motif in ORF57 reduces the dimerization capability of ORF57 and induces protein instability. Thus, we conclude that the zinc ion is important for protein folding and stability of ORF57.

Notably by illustration of first KSHV ORF57 structure, and revealing a novel anti-parallel dimeric fold, a consensus zinc-binding motif, and a novel conformation of the C-terminus which differs from ICP27, our study indicates that, among herpesvirus homologues, different protein in dimerization might have a different mechanism. Perhaps the most important remarkable advance in solving the first ORF57 structure in this study is providing many novel clues for studying ORF57 binding partners and designing small-molecule drugs against KSHV infection and oncogenesis through interfering with ORF57 dimerization and its partner proteins.

## Materials and methods

### Cloning, expression and purification of ORF57

The DNA fragment encoding the residues from 167 to 455 of KSHV ORF57 (GenBank ID: ABD28908.1) was cloned into the pET SUMO vector (Invitrogen, Catalog no. K300-01) in frame with the N-terminal 6×His tag and the SUMO fusion protein. The SUMO (Small Ubiquitin-related Modifier) protein was derived from Smt3 of *Saccharomyces cerevisiae*, and fusing with the SUMO led to increase ORF57-CTD expression and solubility. The protein was expressed in the BL21 (DE3) strain of *Escherichia coli*, supplemented with 50 mg of kanamycin/ml, cultivated at 16°C in Terrific broth for 18 h after induction (at OD_600_ = 0.8) by 0.5 mM IPTG (isopropyl β-D-1-thiogalactopyranoside). For purification, cells were collected at 4°C, 3,500 × *g* for 30 min, the pellet was resuspended in lysis buffer (30 mM Tris-HCl [pH = 8.0], 500 mM NaCl, 1 mM PMSF) and lysed by high-pressure homogenization. After centrifugation at 16,000 × *g* for 40 min at 4°C, the supernatant containing the His-tagged protein was loaded onto immobilized Ni-NTA column (GE Healthcare) pre-equilibrated by binding buffer (30 mM Tris-HCl [pH = 8.0], 500 mM NaCl, 10 mM imidazole), then Ni-NTA affinity column was washed by washing buffer (30 mM Tris-HCl [pH = 8.0], 500 mM NaCl, 120 mM imidazole), finally the recombination protein was eluted by elution buffer (30 mM Tris-HCl [pH = 8.0], 500 mM NaCl, 300 mM imidazole). Afterwards, the ORF57-CTD fusion protein was digested by ULP1 (Ubiquitin-like specific protease 1) overnight, and protein mixture were concentrated by 10 kDa centrifugal filter (Millipore). Then the concentrated recombinant protein was loaded onto gel filtration chromatography (Superdex 75; GE Healthcare) that had been equilibrated in buffer containing 50 mM MES (pH = 6.0), 300 mM NaCl using an Äkta Purifier System (Amersham). The purity of protein was assessed by SDS-PAGE gel and stained by Coomassie brilliant blue, the highest purity fractions were concentrated by 10 kDa centrifugal filter for further crystal screening. The concentration of ORF57-CTD protein was assessed by determining the UV absorbance at 595 nm and by staining with Coomassie Brilliant Blue G250 at a ratio of 1:1000 (Bio-Rad), eventually the ORF57-CTD protein was immediately subject to the following experiments or quickly frozen by liquid nitrogen for long-term storage at -80°C.

### Crystallization, data collection, structure determination and refinement

The ORF57-CTD protein was concentrated to 15 mg/ml and crystallized by sitting-drop vapor diffusion method at 4°C. Crystallization screens were performed by Hampton Research and Qiagen kits. The spindle shaped crystal crystalized in a 1:1 mixture solution of 0.1 M BIS-TRIS propane pH = 7.0, 0.8 M lithium sulfate monohydrate, etc. (Table 1). The crystal size was optimized by gradually changing salt concentration, pH and temperature. The spindle shape crystal had a very poor anisotropic diffraction resolution around 10 Å at room temperature, or under 100 kelvin after rapidly frozen by liquid nitrogen. To improve resolution, the crystals were soaked into different cryo-agents [CryoPro–Cryoprotectant Kit (Hampton Research)], a better diffraction pattern illustrated that soaking crystal in 25% (vol/vol) ethylene glycerol for few seconds to improve diffraction quality from 10 Å to 6 Å. Furthermore, the crystals were kept in the reservoir against the air for several minutes and then soaked into 25% ethylene glycerol before freezing, surprisingly leading to an improved diffraction to 3.5 Å.

Micro seeding was performed to further improve diffraction resolution. The seeds stocking were prepared by grinding crystal on glass and pulverizing crystal to microscopic particles. Microseeds were collected in 10 ul crystallized buffer and diluted to 10^−7^ for a seed working solution. The cubic crystal was obtained by adding 0.1 ul seeds stocking into a 2 ul solution mixture (1 ul protein and 1 ul reservoir), the reservoir contained 0.01 M MgCl_2_·6H_2_O, 0.05 M HEPES sodium pH = 7.0, 4.0 M LiCl.

Both of the crystals were dehydrated by against air for a few minutes and then soaked into cryo-protectant by adding 25% ethylene glycol in reservoir solution for a few seconds and finally flash-frozen in liquid nitrogen for data collection.

Considering the native ORF57-CTD expressed in Terrific broth lacks of phase angels and to solve the phase ambiguity problem, Seleno-methionine (SeMet) was used in basic M9 medium to label ORF57-CTD-SUMO protein and screen for heavy metals in the expression of ORF57-CTD. Surprisingly ORF57-CTD-SUMO expressed highly in the presence of Zn^2+^. Subsequently, Se-labeled ORF57-CTD was successfully crystallized in the conditions for native cubic crystal.

All of the diffraction data were collected at beamline BL17U1 of Shanghai Synchrotron Radiation Facility (SSRF). The monomer (cubic crystal) data set at 3 Å was collected at the peak wavelength of Zn atom (1.2834 Å) and the 3.5 Å dimer (spindle shaped crystal) data set was collected at the wavelength 0.97893 Å. All the data were integrated and scaled with HKL2000. The monomeric structure was solved by single-wavelength anomalous-dispersion (SAD) method using Zn ion as the anomalous scattering atom and then it was used as the searching model to determine the dimeric structure by molecular replacement (MR). Phenix [55] was applied for structure refinement. Structural deviations were corrected manually by Coot [56]. The data collection and refinement statistics are listed in Table 1. Structural figures were produced by Pymol [57].

### Cell lines and plasmids

HEK-293, iSLK-PURO [58], COS-1 (ATCC CRL-1650) cells were grown in Dulbecco's modified Eagle's medium (with 4 mM Glutamine) containing 10% calf serum, 50 IU/ml penicillin, and 50 μg/ml streptomycin (Biological Industries). The 250 μg/ml G418, and 1 μg/ml puromycin (A.G. Scientific) were supplied to iSLK-PURO growth medium to maintain the Tet-On transactivator (Clontech) and RTA expression cassette, respectively. The expression plasmids ORF57-FLAG, ORF57-3×FLAG, ORF57-GFP were generated by inserting ORF57 coding region into pFLAG-CMV-5.1 (Sigma-Aldrich), p3×FLAG-CMV-14 (Sigma-Aldrich) and pEGFP-N1 (Clontech) separately as described [18]. The ORF57-Δ219-FLAG was constructed by inserting ORF57-Δ219 into pFLAG-CMV-5.1 vector. The ORF57-FLAG with the internal deletion of aa167-219 was generated by overlapping PCR and cloned into pFLAG-CMV-5.1 vector. The ORF57-Δ166-GFP and ORF57-Δ219-GFP were constructed by inserting ORF57-Δ166, ORF57-Δ219 into pEGFP-N1 separately. To avoid this GFP fusion transcript splicing from the intron 2 5’ splice site of ORF57 to a cryptic 3’ splice site in the GFP coding region [29] we mutated the intron 2 5’ splice site in ORF57 in all ORF57-GFP expression vectors without affecting the ORF57 protein sequence by overlapping PCR mutagenesis (S1 Table). ORF57-R270A+R271A-FLAG, ORF57-R325A+R327A-FLAG, ORF57-R270A+R271A+R325A+R327A-FLAG were generated by PCR mutagenesis using overlapping primers (S1 Table). The construction of ORF57- W292P-3×FLAG, ORF57- K345A-3×FLAG and ORF57-mt1+2+3 with all three mutated nuclear localization signals was previously described [7,18]. The KSHV-JCS-1 cDNA were purified from JSC-1 cells, the HSV-1-strain F cDNA was a kind gift from Dr. Chun-Fu Zheng of Soochow University, the HCMV-AD169 cDNA was kindly provided by Dr. Zhi-Kang Qian of Institute of Pasteur in China, the EBV-Akata cDNA and MHV68 cDNA was kindly supplied by Dr. Xiao-Zhen Liang of Institute of Pasteur in China. The ORF57-SF plasmid was cloned by inserting ORF57 cDNA into modified pCDH-SF-IRES-Blast vector [59], ICP27-HA, EB2-HA, UL69-HA, mORF57-HA were constructed by amplifying ICP27, EB2, UL69, mORF57 derived from appropriately cDNA described above and inserting into the pCMV-HA vector. All clones with CHCC mutations were performed by overlapping PCR mutagenesis.

### Antibodies and western-blotting analysis

The following primary antibodies were used: anti-FLAG (Sigma-Aldrich, F1804), anti-HA (Sigma-Aldrich, H6908), anti-GFP (Sigma-Aldrich, G1546), anti-GAPDH (Sigma-Aldrich, G8796), anti-β-tubulin (Sigma-Aldrich, T6199), anti-ORF57 antibodies were described previously [18]. The secondary antibodies were as follows: goat anti-mouse IRDye800 (LI-COR, 926–32210), goat anti-rabbit IRDye800 (LI-COR, 926–32211), goat-anti-mouse-horseradish peroxidase (HRP) (Sigma-Aldrich, A2554). All protein samples were denatured by SDS-loading buffer and separated by SDS-polyacrylamide gel and transferred to nitrocellulose (NC) membrane, the individual proteins were stained by appropriate antibodies and detected by ODYSSEY (LI-COR) or SuperSignal West Pico PLUS Chemiluminescent Substrate (Thermo Fisher Scientific).

### Chemical protein cross-linking

Disuccinimidyl suberate (DSS, Thermo Fisher Scientific) was always freshly made by dissolving in DMSO as a 50 mM stock solution and diluted 1:100 with DMSO to working concentration of 500 μM (Thermo Fisher Scientific). Experimental procedures were described previously [18]. Briefly, HEK293 cells plated in a 6-well plate (5×10^5^ cells/well) were transfected with 0.5–2 μg of ORF57 expression vectors. To increase the protein level, in some cases, the cells were treated with 20 μM of proteasome inhibitor MG132 for 4–6 hours before harvesting. Twenty four hours after transfection the cells were harvested, washed with PBS and resuspended in 200 μl of phosphate-buffered saline (PBS). The cross-linking was carried out by addition of 2 μl of 500 μM DSS into 20 μl of cell suspension (50 μM of DSS in final concentration) followed by 30 min incubation at room temperature. Cells treated in parallel with DMSO (vehicle) were used as a negative control. The cross-linking was stopped by quenching of remaining DSS for 15 min by addition of 1 μl of 200 mM Tris-HCl (pH = 7.4). Finally, an equal amount of 2×SDS-loading buffer was added to sample for Western blotting.

### Nuclear translocation assay

COS-1 cells grown on glass cover slips in 6-well plates were transfected with mixture of 0.5 μg of ORF57-GFP and 1.0 μg of ORF57-FLAG expressing vectors. Twenty four hours after transfection the cells were fixed with 2% paraformaldehyde for 30 min, followed by 10 min quenching with 100 mM glycine in PBS and permeabilization with 0.5% Triton X-100 in PBS for 15 min. The slides were subsequently blocked in 3% blot qualified bovine serum albumin (Promega) in PBS containing 0.02% Tween-20 (blocking solution), and incubated with anti-FLAG M2 primary antibody (F3165, Sigma-Aldrich) diluted in blocking buffer for 2 h at 37°C and further stained with anti-mouse Alexa Fluor 594 secondary antibody in blocking buffer for 30 min at 37°C. Cell nuclei were stained for 15 min at RT with Hoechst 33342 (Thermo Fisher Scientific). Slides were washed, mounted and captured with a fluorescence microscope (Olympus). The nuclear translocation frequency was determined by observation of 50 ORF57-GFP/ORF57-FLAG double positive cells. A positive translocation was defined by the cells exhibiting higher, nuclear GFP signal intensity over the cytoplasmic count.

### Protein stability and half-life assays

To inhibit proteasome-mediated degradation, the HEK293 cells in 6-well plate (5×10^5^ cells/well) were transfected in parallel with ORF57 wt or mutant expression vectors. Twenty hours after transfection the cells were treated with proteasome inhibitor MG132 (20 μM final) for additional 4 hours. The cells treated with DMSO (vehicle) were used as negative control. To determine the half-life of the wt ORF57 or CHCC mutant protein, HEK293 cells in 6-well plate (5×10^5^ cells/well) were transfected with 0.5 μg of wt ORF57 or 2 μg of CHCC mutant expression vector. Forty hours after transfection, the cells were treated with 50 μM CHX in final concentration to inhabit protein synthesis and the cell lysates were collected at the indicated time for Western blot. The half-life (t_1/2_) of wt ORF57 or CHCC mutant protein was determined by a line plot analysis as described [60].

### Northern blotting analysis

HEK-293 cells transfected with 2 μg plasmids were harvested 24h after transfection and lysed in TRIzol reagent (Invitrogen). Total RNA samples were separated in 1% agarose and transferred onto nylon membrane, hybridization was performed using a ^32^P-labeled probe (S1 Table) of ORF57 as described [7].

### Construction of BAC16 ORF57 mutant virus

The KSHV genome was modified by using a two-step “scarless” homologous recombination procedure in GS1783 *E*. *coli* strain harboring BACmid containing full-length KSHV genome that was described previously [32]. For CHCC mutant virus, the cysteine and histidine were replaced by serine and leucine, respectively. The Kan^r^ I-SceI cassette was amplified from the pEP-Kan-S plasmid using primers described in S2 Table. The first round inserted two mutation sites, the second and third rounds inserted one mutation site separately. The recombination procedure was performed as previous described [32]. The selected clone were sequenced and amplified.

### BAC DNA isolation and analysis

BAC16 was isolated by NucleoBond Xtra Midi kit (MACHEREY-NAGEL), modified regions were PCR amplified and sequenced. To further verify the integrity of recombinant BACmids, the purified BAC DNA was digested with *XhoI* and separated on a 0.8% agarose gel in 0.5 × TAE under the following conditions: 80 V/cm for 4 h.

### Transfection of KSHV BAC16 and establishment of stable cell line

The iSLK-PURO cells were grown to ∼80% confluence in a 6-well plate followed by transfection with 10 ug of BAC DNA using FuGENE HD Transfection Reagent (Promega). Cells were selected after 48 hours in a medium which contained 250 μg/ml G418 (Sigma-Aldrich), 1 μg/ml puromycin (Sigma-Aldrich), 1000 μg/ml hygromycin B (Millipore). Three weeks later, iSLK-PURO cells infected with recombinant virus were established.

### RNA isolation and qPCR

iSLK-PURO cells were treated with DOX (Clontech) to induce RTA expression and KSHV lytic cycle reactivation. To quantify virus genes expression, total RNA was extracted with Tri-reagent (Sigma-Aldrich) according to manufacturer's instructions. 0.5 ug of total RNA was reverse transcribed by using PrimeScript RT reagent Kit with gDNA Eraser, cDNA was quantified by using SYBR Fast qPCR Mix (Takara) and operated on LightCycler 480II (Roche). The relative quantification of gene expression was calculated by using the comparative threshold cycle (CT) method (2^−ΔΔ^ CT), cellular level of GAPDH RNA was used as a reference. The transcription level of wild type and mutant genes of ORF57, UL69, EB2, mORF57 were detected by the same way. The qPCR primers are listed in S3 Table.

### Virus replication and production assay

iSLK-wt and CHCC mutant cells were induced with 1ug/ml of DOX and incubated at 37°C for 72, 96 and 120 h, the KSHV genome was purified from the supernatant (200 ul of 2×10^6^ cells) by TIANamp Blood DNA Kit (TIANGEN) and quantified by qPCR using K9 primers (S3 Table) for KSHV copy numbers. The supernatant (500ul) of each group at 72 hours was incubated with HEK293T cells plated in 6-well plate for 24 hours, and the percentage of GFP positive cells were calculated by FACS assay.

### Accession numbers

The accession numbers for the crystal structures of ORF57-dimer and ORF57-monomer have been deposited in the Protein Data Bank (accession numbers are 5zb3 and 5zb1, respectively).

## Supporting information

S1 Fig The shapes of ORF57-CTD crystals and data collection. (A) The images of ORF57-CTD crystals were captured by ADSC Q315 detector. (B) The typical X-ray diffraction image.
(PPTX)

S2 Fig Superimposition of the monomeric ORF57-CTD in dimer and monomer visualized in PyMol. (PPTX)

S3 Fig **The clustered hydrophobic residues in the N-terminal arm (A) and C-terminal end (B) of ORF57-CTD.** (A) The residues in N-terminal arm in a stick model display hydrophobicity in scaled color in PyMol scripts, with the highly hydrophobic residues labeled in black letters. (B) The C-terminal end is rich in hydrophobic residues in scaled color by PyMol scripts. The interactions between the hydrophobic C-terminal end (black) and the surrounding residues (green) are shown in black dash lines with the distances between the interacting atoms showed in Å.
(PPTX)

S4 Fig Conservation of the C-terminal end among herpesviral homologs. The C-terminal end (F445-L454) is in a medium degree of conservation (scaled color in PyMol with the ConSurf Server).
(PPTX)

S5 Fig Comparison of polar intermolecular interactions within ORF57 and ICP27 dimer. The diagrams illustrates the polar intermolecular interactions between “arm” (green box) and globular (yellow box) domains (a) and between two globular domains (b) in the ORF57 dimer and ICP27 dimer (PDB ID: 4yxp).The numbered yellow boxes represent individual α-helixes. The dash lines of ORF57 and ICP27 show hydrogen bonds (blue lines) or salt bridges (red lines) between interacting residues. Interface interaction analyses of ORF57 and ICP27 were done by using PDBe-PISA (http://www.ebi.ac.uk/msd-srv/prot_int/cgi-bin/piserver) and the interface interaction residues of ORF57 are also listed in Supplemental Table S4.
(PPTX)

S6 Fig Deletion of the “arm” region leads to protein instability. (A and B) Deletion of the “arm” region from ORF57-CTD affects the stability of ORF57 protein. HEK293 cells were transfected with FLAG-tagged full-length ORF57 (full) or Δ219 mutant (Δ219). At 18 h post transfection, the cells were treated with a proteasome inhibitor MG132 or DMSO (vehicle), for additional 6 h and ORF57 expression was analyzed by Western blotting using an anti-FLAG antibody (A). Total RNA isolated from the cell lysates in parallel was digested by DNase I and examined by RT-PCR for the overall level of transcribed ORF57 RNA from individual expression vectors (B). The RNA samples minus RT (lanes 1, 3 and 5) were controls for possible residual DNA contamination. The water (lane 7) and vector DNA (lane 8) were used as controls (B). (C) Expression of GFP-tagged ORF57 and truncation mutants in HEK293. Cells transfected with indicated expression vectors were harvested at 24 h post transfection and the cell lysates were analyzed for ORF57 protein expression by Western blotting using anti-GFP antibody.
(PPTX)

S7 Fig The dimerization activities of wild type (WT) and mutant ORF57 in nuclear translocation assays. The wider area from nuclear translocation assays showed in Figs 5E and 6C with the double ORF57-GFP-positive/ORF57-FLAG-positive (yellow arrow) and single ORF57-GFP-positive/ORF57-FLAG-negative (white arrows, no ORF57-FLAG expression) in the same microscopic field.
(PPTX)

S8 Fig Structure-based sequence alignment of KSHV ORF57 and its homologues. Multiple alignment of the protein sequences was performed by Clustal Omega for ICP27 (herpes simplex virus type 1 and type 2, HSV1-ICP27 and HSV2-ICP27), ORF4 (varicella-zoster virus, VZV-ORF4), EB2 (Epstein-Barr virus, EBV-EB2), UL69 (human cytomegalovirus, HCMV-UL69), and mORF57 (murine gamma herpesvirus 68, MHV68-mORF57), with the conserved residues in red surrounded by blue boxes, identical residues in red, and the residues of the zinc-binding motif in red stars. The secondary structural elements of ORF57-CTD were analyzed by ESPript3 (http://espript.ibcp.fr/ESPript/cgi-bin/ESPript.cgi), with the indicated α-helix (coil), η-helix (coil), β-sheet (arrows), and turn (T) above the alignment.
(PPTX)

S9 Fig The Zinc-binding motif-defective ORF57 protein (CHCC Mut) has a shorter half-life than wild-type (WT) ORF57 protein. (A) HEK293 cells were transfected with KSHV WT ORF57 or CHCC mutant expression vectors for 40 h and then incubated with 50 μM CHX for the indicated time. The expression level of ORF57 were detected with an anti-Flag antibody. GAPDH served as a loading control. (B) The protein half life of ORF57 WT or CHCC mutant was calculated based on the amount of remaining ORF57 protein at each time point after normalization to GAPDH.
(PPTX)

S10 Fig Construction of KSHV ORF57 zinc-binding motif mutant virus. The genome of the mutant virus (C333S/H423L/C427S/C432S) was constructed by two step scarless recombination in BAC16. (A) Sequencing results of the recombinant BAC16 confirmed the introduced four mutations. (B) The integrity of recombinant BAC16 was digested with XhoI and the digestion products were separated on a 0.8% agarose gel (lane 1: WT; lane 2: CHCC mutant; lane 3: 1 Kb DNA ladder). The DNA bands bearing ORF57 segments are marked with red triangles.
(PPTX)

S11 Fig The intermolecular interactions of E287, E288, W292, K345 to the surrounding residues in the ORF57 globular domain. (A) The α-helix 4 (yellow) inserts into the core region of the globular domain (left two panels). The residues E287, E288, W292 in α-helix 4 mediate a large number of interactions (black dashed lines) with the surrounding residues (highlighted in cyans), leading to structure stabilization (right panel). (B) The residue K345 protrudes out from the surface of ORF57-CTD and does not form any interaction with other residues.
(PPTX)

S12 Fig The shape and surface electrostatic charges of ORF57-CTD differ from that of ICP27-CTD. (A and B) The shape and electrostatic calculations of a monomeric structure in dimerized ORF57-CTD (A) and ICP27-CTD (B, PDB ID: 4yxp) calculated by PyMol with the APBS with180˚ rotation, red, negative charge; blue, positive charge.
(PPTX)

S1 Movie Crystal structure of ORF57 monomer with 3Å resolution showed as a cartoon in rainbow spectrum coloring with dark blue for the N-terminus and red color for the C-terminus. The black sphere marks the zinc atom.
(MP4)

S2 Movie Crystal structure of ORF57 dimer with 3.5Å resolution. One monomer is showed as a cartoon in rainbow spectrum coloring with dark blue for the N-terminus and red color for the C-terminus. The second monomer is colored in grey. The black spheres mark the zinc atoms in each monomer.
(MP4)

S1 Table Cloning primers and Northern probe sequences. (DOCX)

S2 Table BAC16 mutagenesis primers. (DOCX)

S3 Table qPCR primers. (DOCX)

S4 Table PDBePISA analysis of interactions in the dimer interface. (DOCX)

S1 File PDB validation report of the monomer structure. (PDF)

S2 File PDB validation report of the dimer structure. (PDF)
