# Supplementary material for: The crystal structure of KSHV ORF57 reveals dimeric active sites important for protein stability and function
Source: PLoS Pathog. 2018 Aug 10;14(8):e1007232. doi: 10.1371/journal.ppat.1007232 (PMC6105031; doi:10.1371/journal.ppat.1007232)
Supplement: S1 Fig — (A) The images of ORF57-CTD crystals were captured by ADSC Q315 detector. (B) The typical X-ray diffraction image. (PPTX) [file ppat.1007232.s001.pptx]

## Slide 1
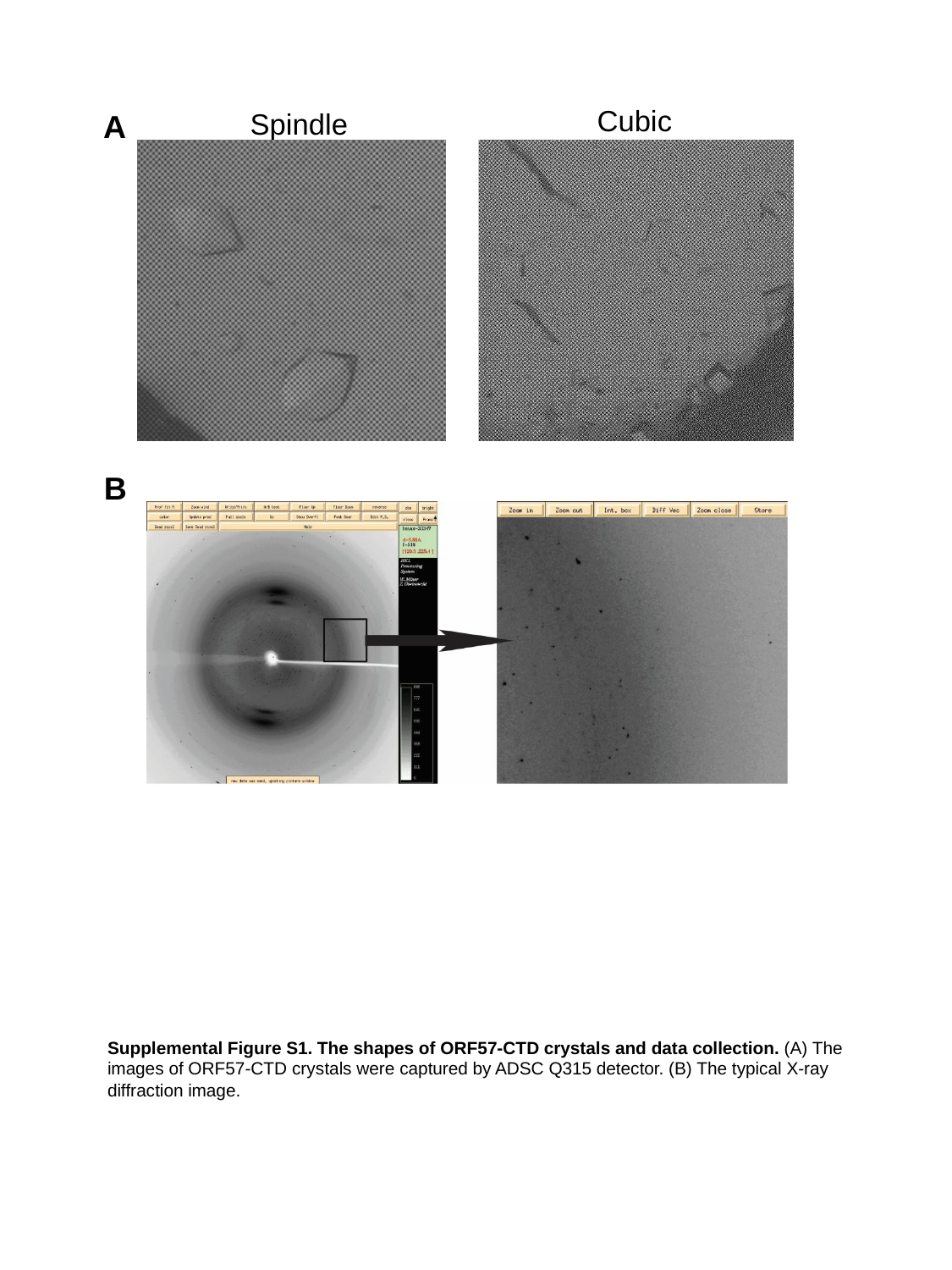

Cubic
Spindle
A
B
Supplemental Figure S1. The shapes of ORF57-CTD crystals and data collection. (A) The images of ORF57-CTD crystals were captured by ADSC Q315 detector. (B) The typical X-ray diffraction image.
