# Supplementary material for: The crystal structure of KSHV ORF57 reveals dimeric active sites important for protein stability and function
Source: PLoS Pathog. 2018 Aug 10;14(8):e1007232. doi: 10.1371/journal.ppat.1007232 (PMC6105031; doi:10.1371/journal.ppat.1007232)
Supplement: S2 Fig — (PPTX) [file ppat.1007232.s002.pptx]

## Slide 1
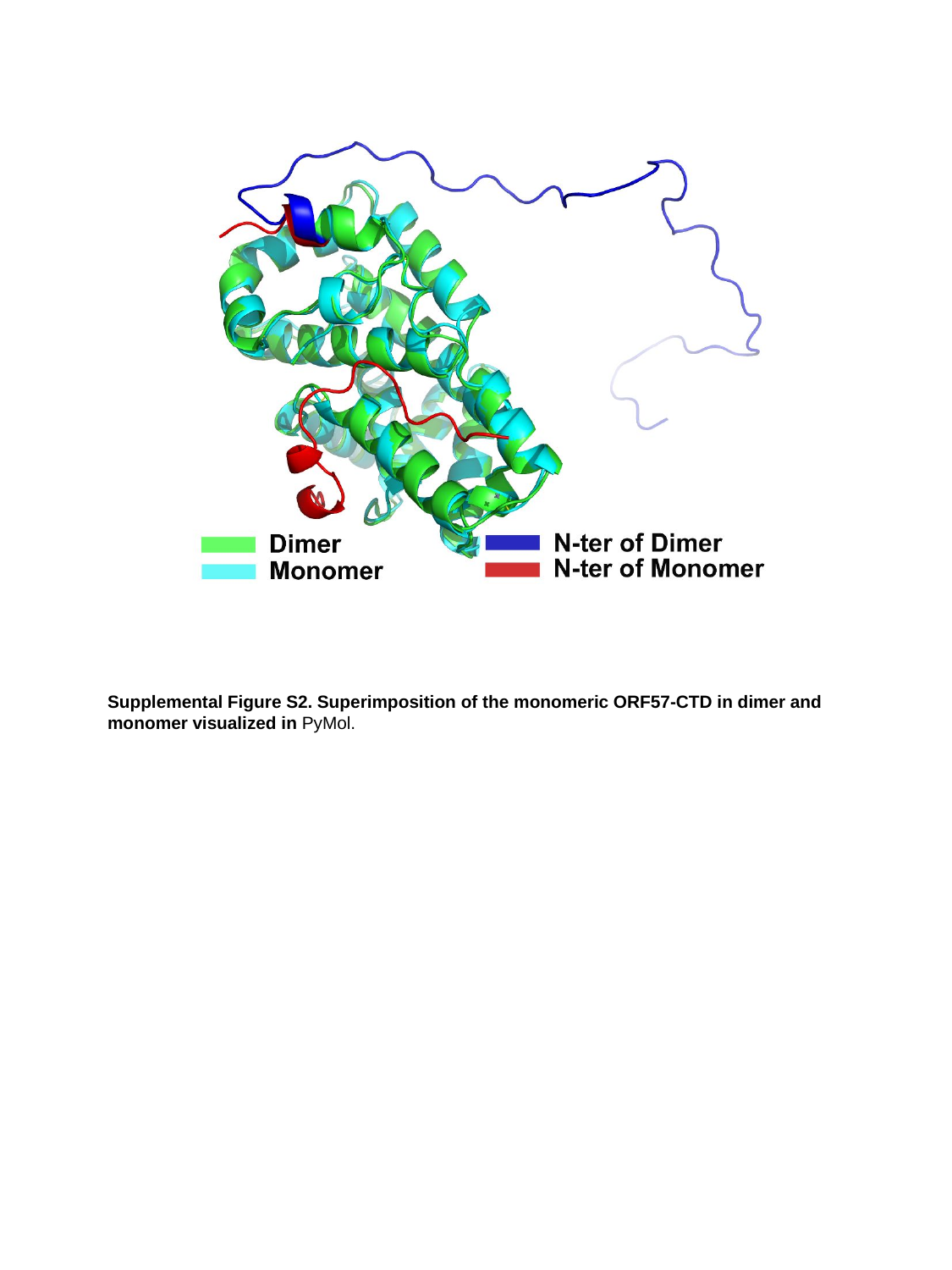

Supplemental Figure S2. Superimposition of the monomeric ORF57-CTD in dimer and monomer visualized in PyMol.
