# Supplementary material for: The crystal structure of KSHV ORF57 reveals dimeric active sites important for protein stability and function
Source: PLoS Pathog. 2018 Aug 10;14(8):e1007232. doi: 10.1371/journal.ppat.1007232 (PMC6105031; doi:10.1371/journal.ppat.1007232)
Supplement: S3 Fig — The clustered hydrophobic residues in the N-terminal arm (A) and C-terminal end (B) of ORF57-CTD. (A) The residues in N-terminal arm in a stick model display hydrophobicity in scaled color in PyMol scripts, with the highly hydrophobic residues labeled in black letters. (B) The C-terminal end is rich in hydrophobic residues in scaled color by PyMol scripts. The interactions between the hydrophobic C-terminal end (black) and the surrounding residues (green) are shown in black dash lines with the distances between the interacting atoms showed in Å. (PPTX) [file ppat.1007232.s003.pptx]

## Slide 1
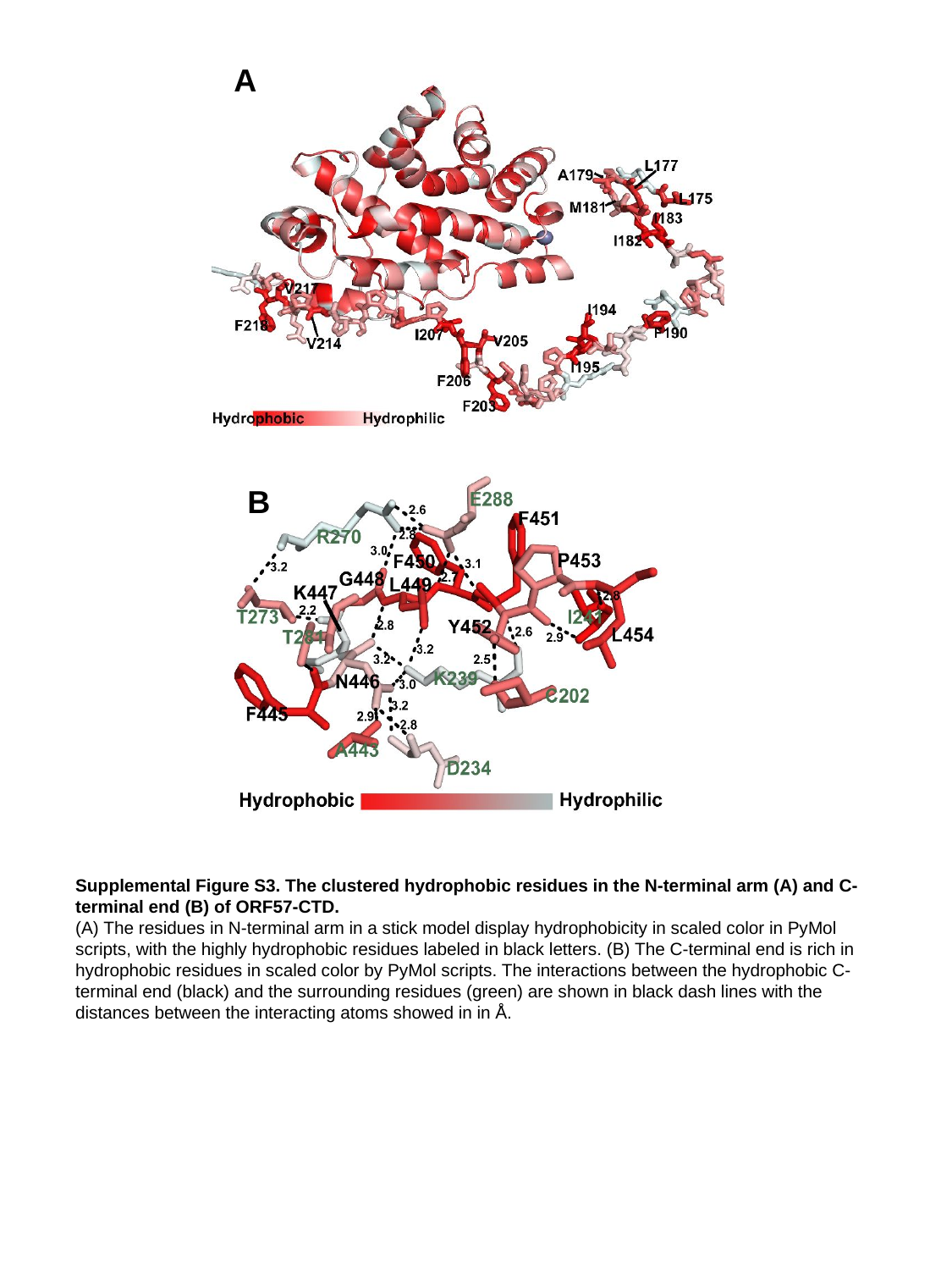

A
B
Supplemental Figure S3. The clustered hydrophobic residues in the N-terminal arm (A) and C-terminal end (B) of ORF57-CTD.
(A) The residues in N-terminal arm in a stick model display hydrophobicity in scaled color in PyMol scripts, with the highly hydrophobic residues labeled in black letters. (B) The C-terminal end is rich in hydrophobic residues in scaled color by PyMol scripts. The interactions between the hydrophobic C-terminal end (black) and the surrounding residues (green) are shown in black dash lines with the distances between the interacting atoms showed in in Å.
