# Supplementary material for: The crystal structure of KSHV ORF57 reveals dimeric active sites important for protein stability and function
Source: PLoS Pathog. 2018 Aug 10;14(8):e1007232. doi: 10.1371/journal.ppat.1007232 (PMC6105031; doi:10.1371/journal.ppat.1007232)
Supplement: S4 Fig — The C-terminal end (F445-L454) is in a medium degree of conservation (scaled color in PyMol with the ConSurf Server). (PPTX) [file ppat.1007232.s004.pptx]

## Slide 1
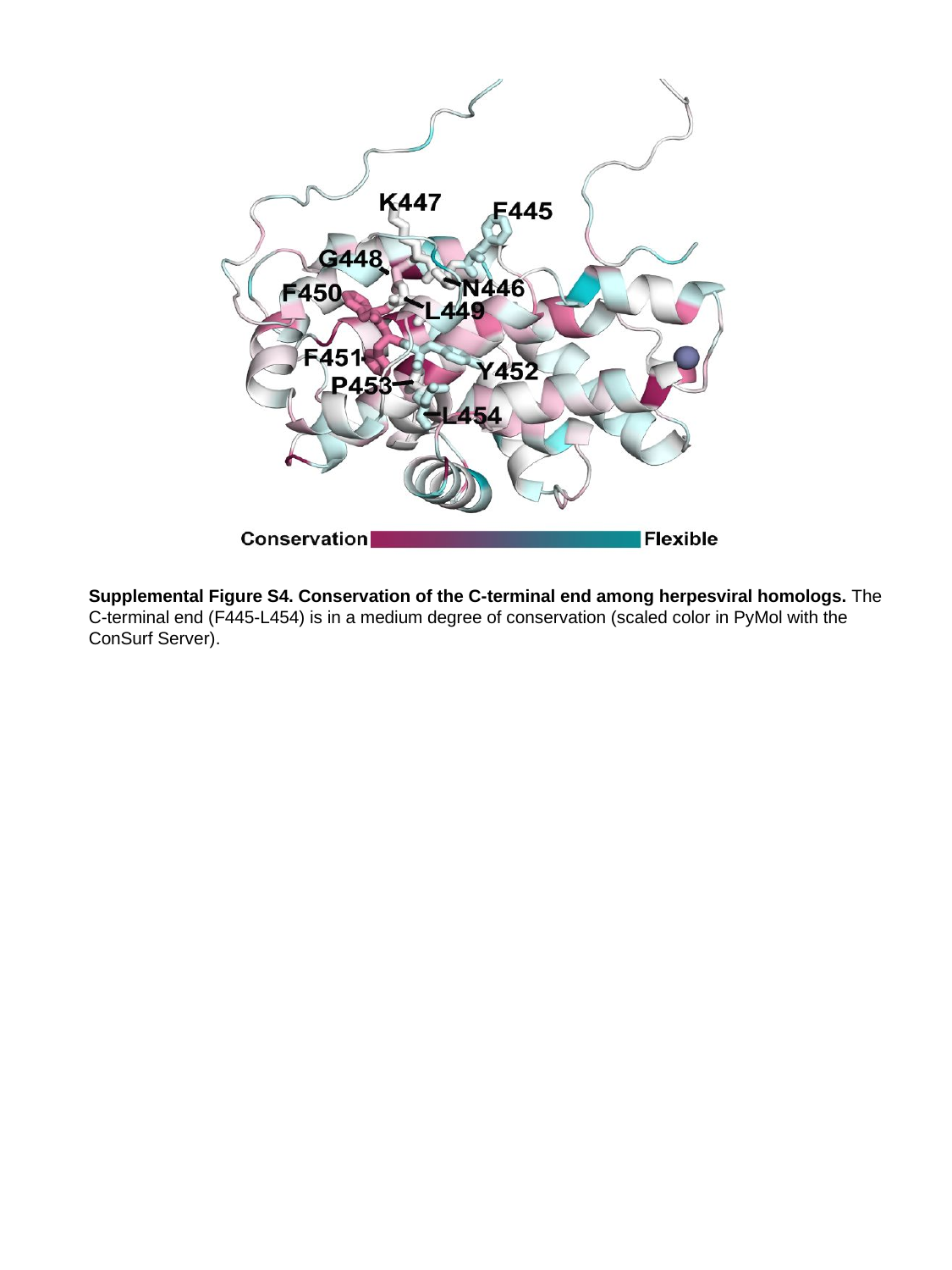

Supplemental Figure S4. Conservation of the C-terminal end among herpesviral homologs. The C-terminal end (F445-L454) is in a medium degree of conservation (scaled color in PyMol with the ConSurf Server).
