# Supplementary material for: The crystal structure of KSHV ORF57 reveals dimeric active sites important for protein stability and function
Source: PLoS Pathog. 2018 Aug 10;14(8):e1007232. doi: 10.1371/journal.ppat.1007232 (PMC6105031; doi:10.1371/journal.ppat.1007232)
Supplement: S5 Fig — The diagrams illustrates the polar intermolecular interactions between “arm” (green box) and globular (yellow box) domains (a) and between two globular domains (b) in the ORF57 dimer and ICP27 dimer (PDB ID: 4yxp).The numbered yellow boxes represent individual α-helixes. The dash lines of ORF57 and ICP27 show hydrogen bonds (blue lines) or salt bridges (red lines) between interacting residues. Interface interaction analyses of ORF57 and ICP27 were done by using PDBe-PISA (http://www.ebi.ac.uk/msd-srv/prot_int/cgi-bin/piserver) and the interface interaction residues of ORF57 are also listed in Supplemental Table S4. (PPTX) [file ppat.1007232.s005.pptx]

## Slide 1
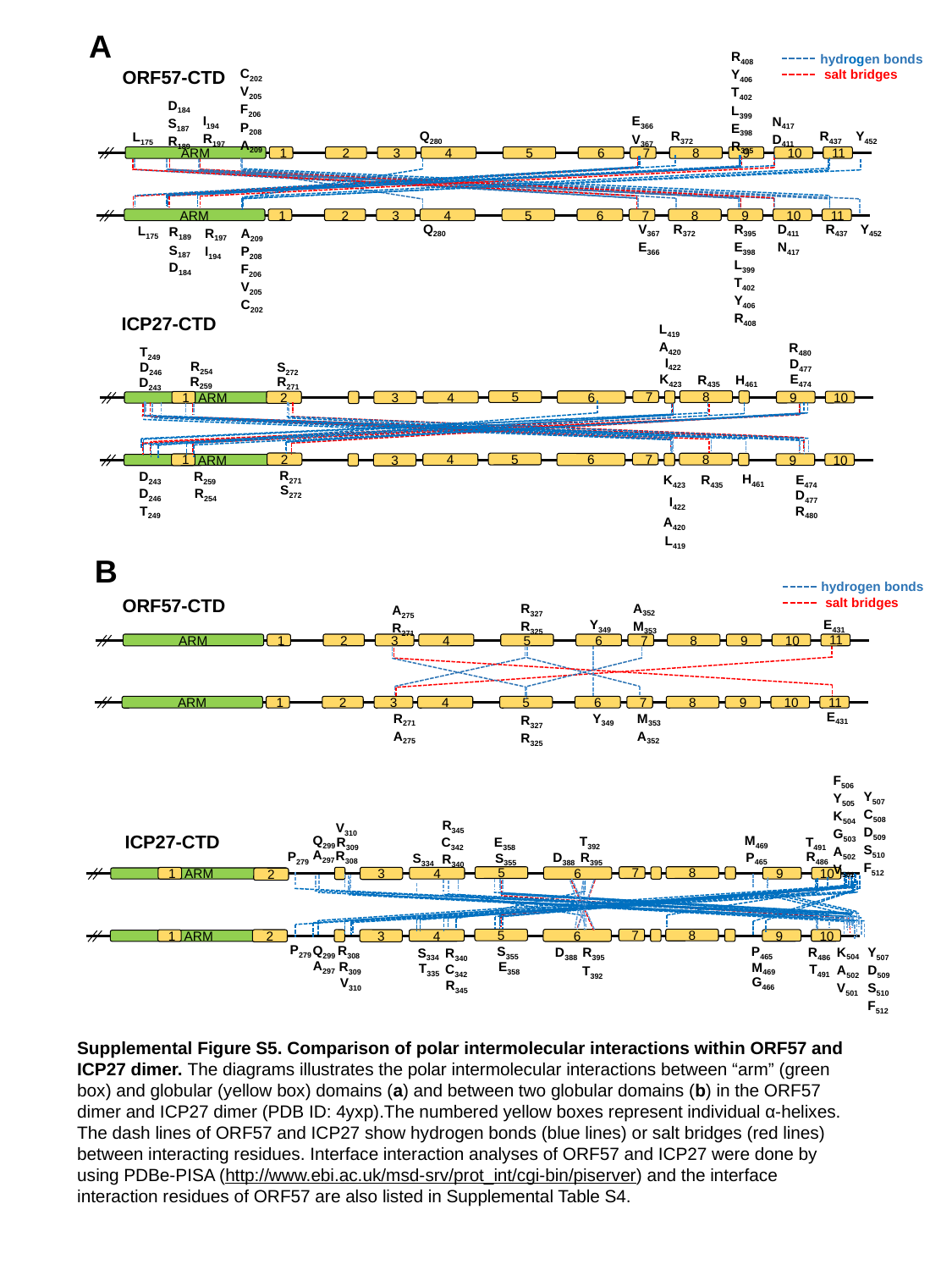

A
hydrogen bonds
C202
V205
F206
P208
A209
salt bridges
D184
S187
R189
I194
R197
L175
10
11
ARM
1
2
3
4
5
6
7
8
9
10
11
ARM
1
2
3
4
5
6
7
8
9
Q280
V367
E366
R372
R395
E398
L399
T402
Y406
R408
D411
N417
R437
Y452
ORF57-CTD
R408
Y406
T402
L399
E398
R395
E366
V367
N417
D411
R437
R372
Y452
Q280
L175
R189
S187
D184
R197
I194
A209
P208
F206
V205
C202
ICP27-CTD
L419
A420
R480
T249
I422
D477
R254
S272
D246
K423
E474
H461
R435
R259
R271
D243
9
10
5
7
8
6
2
3
4
1
ARM
9
10
2
5
7
8
6
3
4
1
ARM
R271
R259
D243
H461
R435
E474
K423
S272
R254
D246
D477
I422
R480
T249
A420
L419
B
hydrogen bonds
ORF57-CTD
salt bridges
A352
M353
R327
R325
A275
R271
Y349
E431
11
10
ARM
1
2
3
4
5
6
7
8
9
10
11
ARM
1
2
3
4
5
6
7
8
9
E431
Y349
R271
A275
M353
A352
R327
R325
F506
Y505
K504
G503
A502
V501
Y507
C508
D509
S510
F512
R345
V310
ICP27-CTD
M469
Q299
T392
R309
E358
C342
T491
A297
R308
P279
R486
P465
D388
R395
S355
S334
R340
9
10
5
7
8
6
3
4
1
ARM
2
9
10
5
7
8
6
2
3
4
1
ARM
P279
Q299
R308
S355
P465
K504
A502
V501
D388
R486
Y507
D509
S510
F512
R395
S334
R340
A297
E358
R309
M469
T335
T491
C342
T392
G466
V310
R345
Supplemental Figure S5. Comparison of polar intermolecular interactions within ORF57 and ICP27 dimer. The diagrams illustrates the polar intermolecular interactions between “arm” (green box) and globular (yellow box) domains (a) and between two globular domains (b) in the ORF57 dimer and ICP27 dimer (PDB ID: 4yxp).The numbered yellow boxes represent individual α-helixes. The dash lines of ORF57 and ICP27 show hydrogen bonds (blue lines) or salt bridges (red lines) between interacting residues. Interface interaction analyses of ORF57 and ICP27 were done by using PDBe-PISA (http://www.ebi.ac.uk/msd-srv/prot_int/cgi-bin/piserver) and the interface interaction residues of ORF57 are also listed in Supplemental Table S4.
