# Supplementary material for: The crystal structure of KSHV ORF57 reveals dimeric active sites important for protein stability and function
Source: PLoS Pathog. 2018 Aug 10;14(8):e1007232. doi: 10.1371/journal.ppat.1007232 (PMC6105031; doi:10.1371/journal.ppat.1007232)
Supplement: S6 Fig — (A and B) Deletion of the “arm” region from ORF57-CTD affects the stability of ORF57 protein. HEK293 cells were transfected with FLAG-tagged full-length ORF57 (full) or Δ219 mutant (Δ219). At 18 h post transfection, the cells were treated with a proteasome inhibitor MG132 or DMSO (vehicle), for additional 6 h and ORF57 expression was analyzed by Western blotting using an anti-FLAG antibody (A). Total RNA isolated from the cell lysates in parallel was digested by DNase I and examined by RT-PCR for the overall level of transcribed ORF57 RNA from individual expression vectors (B). The RNA samples minus RT (lanes 1, 3 and 5) were controls for possible residual DNA contamination. The water (lane 7) and vector DNA (lane 8) were used as controls (B). (C) Expression of GFP-tagged ORF57 and truncation mutants in HEK293. Cells transfected with indicated expression vectors were harvested at 24 h post transfection and the cell lysates were analyzed for ORF57 protein expression by Western blotting using anti-GFP antibody. (PPTX) [file ppat.1007232.s006.pptx]

## Slide 1
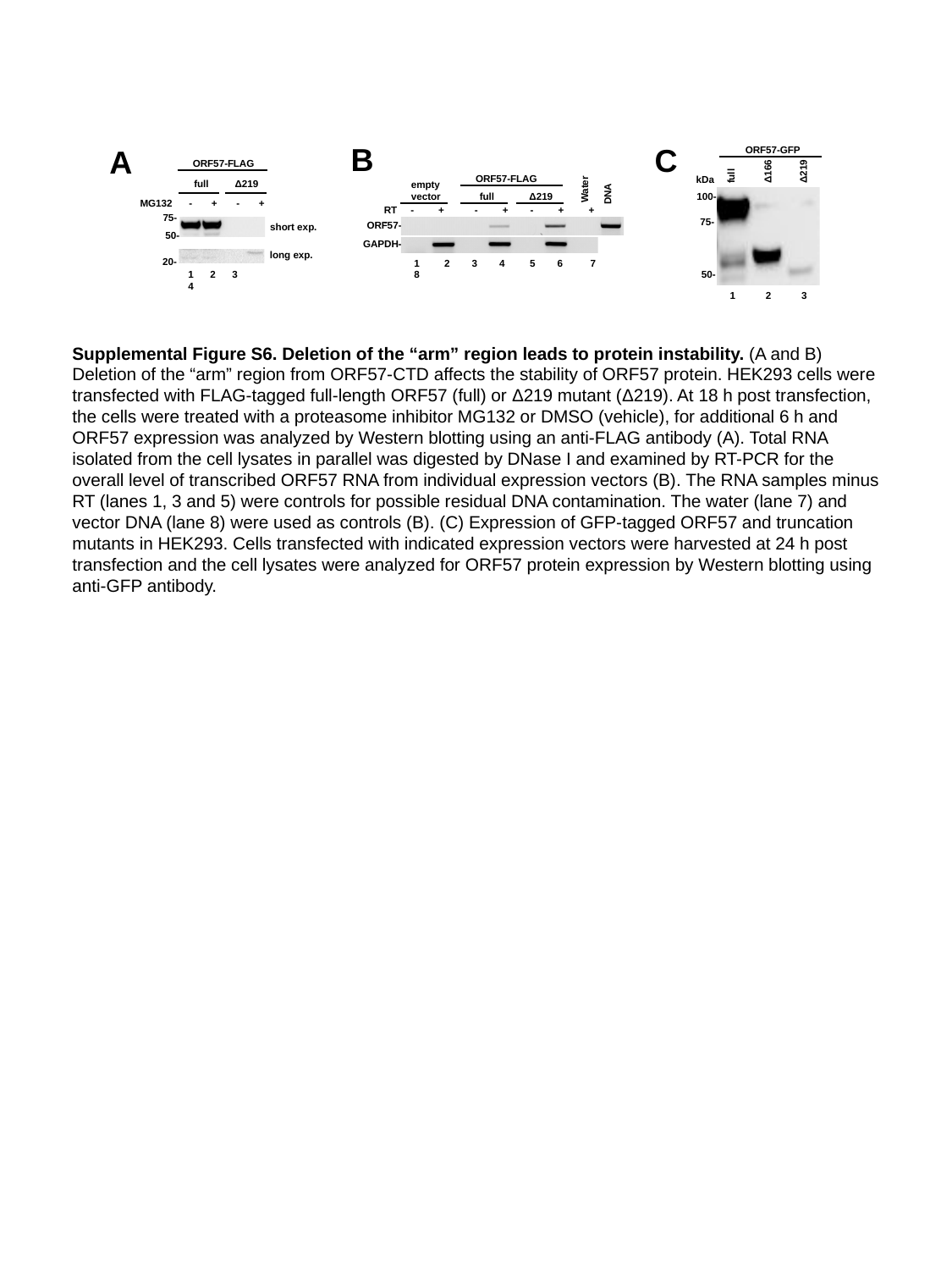

B
C
A
ORF57-GFP
Δ166
Δ219
full
kDa
100-
75-
50-
1 2 3
ORF57-FLAG
full
Δ219
MG132 - + - +
75-
short exp.
50-
long exp.
20-
1 2 3 4
ORF57-FLAG
empty
vector
Water
DNA
full
Δ219
RT - + - + - + + -
ORF57-
GAPDH-
1 2 3 4 5 6 7 8
Supplemental Figure S6. Deletion of the “arm” region leads to protein instability. (A and B) Deletion of the “arm” region from ORF57-CTD affects the stability of ORF57 protein. HEK293 cells were transfected with FLAG-tagged full-length ORF57 (full) or Δ219 mutant (Δ219). At 18 h post transfection, the cells were treated with a proteasome inhibitor MG132 or DMSO (vehicle), for additional 6 h and ORF57 expression was analyzed by Western blotting using an anti-FLAG antibody (A). Total RNA isolated from the cell lysates in parallel was digested by DNase I and examined by RT-PCR for the overall level of transcribed ORF57 RNA from individual expression vectors (B). The RNA samples minus RT (lanes 1, 3 and 5) were controls for possible residual DNA contamination. The water (lane 7) and vector DNA (lane 8) were used as controls (B). (C) Expression of GFP-tagged ORF57 and truncation mutants in HEK293. Cells transfected with indicated expression vectors were harvested at 24 h post transfection and the cell lysates were analyzed for ORF57 protein expression by Western blotting using anti-GFP antibody.
