# Supplementary material for: The crystal structure of KSHV ORF57 reveals dimeric active sites important for protein stability and function
Source: PLoS Pathog. 2018 Aug 10;14(8):e1007232. doi: 10.1371/journal.ppat.1007232 (PMC6105031; doi:10.1371/journal.ppat.1007232)
Supplement: S7 Fig — The wider area from nuclear translocation assays showed in Figs 5E and 6C with the double ORF57-GFP-positive/ORF57-FLAG-positive (yellow arrow) and single ORF57-GFP-positive/ORF57-FLAG-negative (white arrows, no ORF57-FLAG expression) in the same microscopic field. (PPTX) [file ppat.1007232.s007.pptx]

## Slide 1
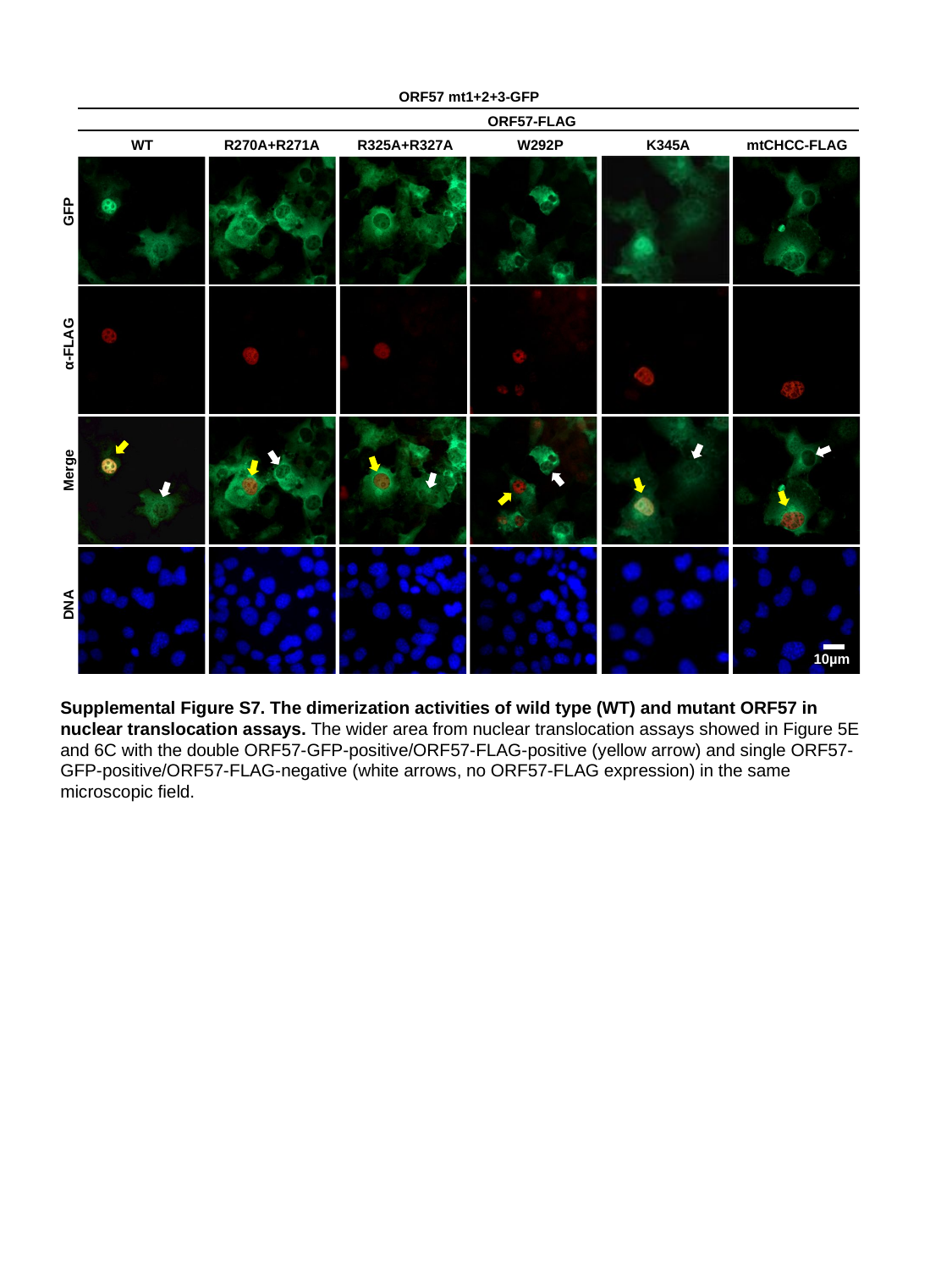

ORF57 mt1+2+3-GFP
ORF57-FLAG
WT
R270A+R271A
R325A+R327A
W292P
K345A
mtCHCC-FLAG
GFP
α-FLAG
Merge
DNA
10µm
Supplemental Figure S7. The dimerization activities of wild type (WT) and mutant ORF57 in nuclear translocation assays. The wider area from nuclear translocation assays showed in Figure 5E and 6C with the double ORF57-GFP-positive/ORF57-FLAG-positive (yellow arrow) and single ORF57-GFP-positive/ORF57-FLAG-negative (white arrows, no ORF57-FLAG expression) in the same microscopic field.
