# Supplementary material for: The crystal structure of KSHV ORF57 reveals dimeric active sites important for protein stability and function
Source: PLoS Pathog. 2018 Aug 10;14(8):e1007232. doi: 10.1371/journal.ppat.1007232 (PMC6105031; doi:10.1371/journal.ppat.1007232)
Supplement: S8 Fig — Multiple alignment of the protein sequences was performed by Clustal Omega for ICP27 (herpes simplex virus type 1 and type 2, HSV1-ICP27 and HSV2-ICP27), ORF4 (varicella-zoster virus, VZV-ORF4), EB2 (Epstein-Barr virus, EBV-EB2), UL69 (human cytomegalovirus, HCMV-UL69), and mORF57 (murine gamma herpesvirus 68, MHV68-mORF57), with the conserved residues in red surrounded by blue boxes, identical residues in red, and the residues of the zinc-binding motif in red stars. The secondary structural elements of ORF57-CTD were analyzed by ESPript3 (http://espript.ibcp.fr/ESPript/cgi-bin/ESPript.cgi), with the indicated α-helix (coil), η-helix (coil), β-sheet (arrows), and turn (T) above the alignment. (PPTX) [file ppat.1007232.s008.pptx]

## Slide 1
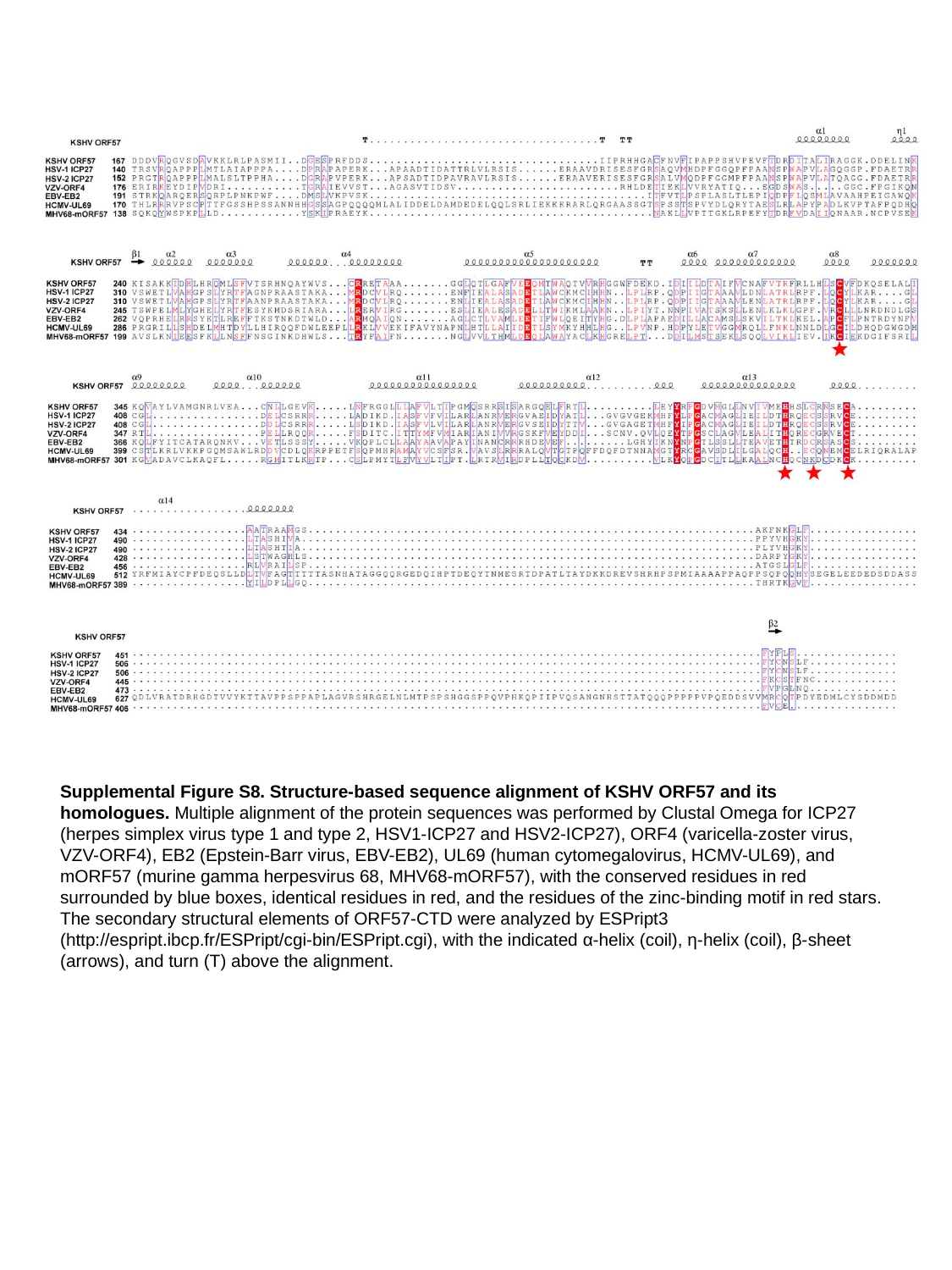

Supplemental Figure S8. Structure-based sequence alignment of KSHV ORF57 and its homologues. Multiple alignment of the protein sequences was performed by Clustal Omega for ICP27 (herpes simplex virus type 1 and type 2, HSV1-ICP27 and HSV2-ICP27), ORF4 (varicella-zoster virus, VZV-ORF4), EB2 (Epstein-Barr virus, EBV-EB2), UL69 (human cytomegalovirus, HCMV-UL69), and mORF57 (murine gamma herpesvirus 68, MHV68-mORF57), with the conserved residues in red surrounded by blue boxes, identical residues in red, and the residues of the zinc-binding motif in red stars. The secondary structural elements of ORF57-CTD were analyzed by ESPript3 (http://espript.ibcp.fr/ESPript/cgi-bin/ESPript.cgi), with the indicated α-helix (coil), η-helix (coil), β-sheet (arrows), and turn (T) above the alignment.
