# Supplementary material for: The crystal structure of KSHV ORF57 reveals dimeric active sites important for protein stability and function
Source: PLoS Pathog. 2018 Aug 10;14(8):e1007232. doi: 10.1371/journal.ppat.1007232 (PMC6105031; doi:10.1371/journal.ppat.1007232)
Supplement: S9 Fig — (A) HEK293 cells were transfected with KSHV WT ORF57 or CHCC mutant expression vectors for 40 h and then incubated with 50 μM CHX for the indicated time. The expression level of ORF57 were detected with an anti-Flag antibody. GAPDH served as a loading control. (B) The protein half life of ORF57 WT or CHCC mutant was calculated based on the amount of remaining ORF57 protein at each time point after normalization to GAPDH. (PPTX) [file ppat.1007232.s009.pptx]

## Slide 1
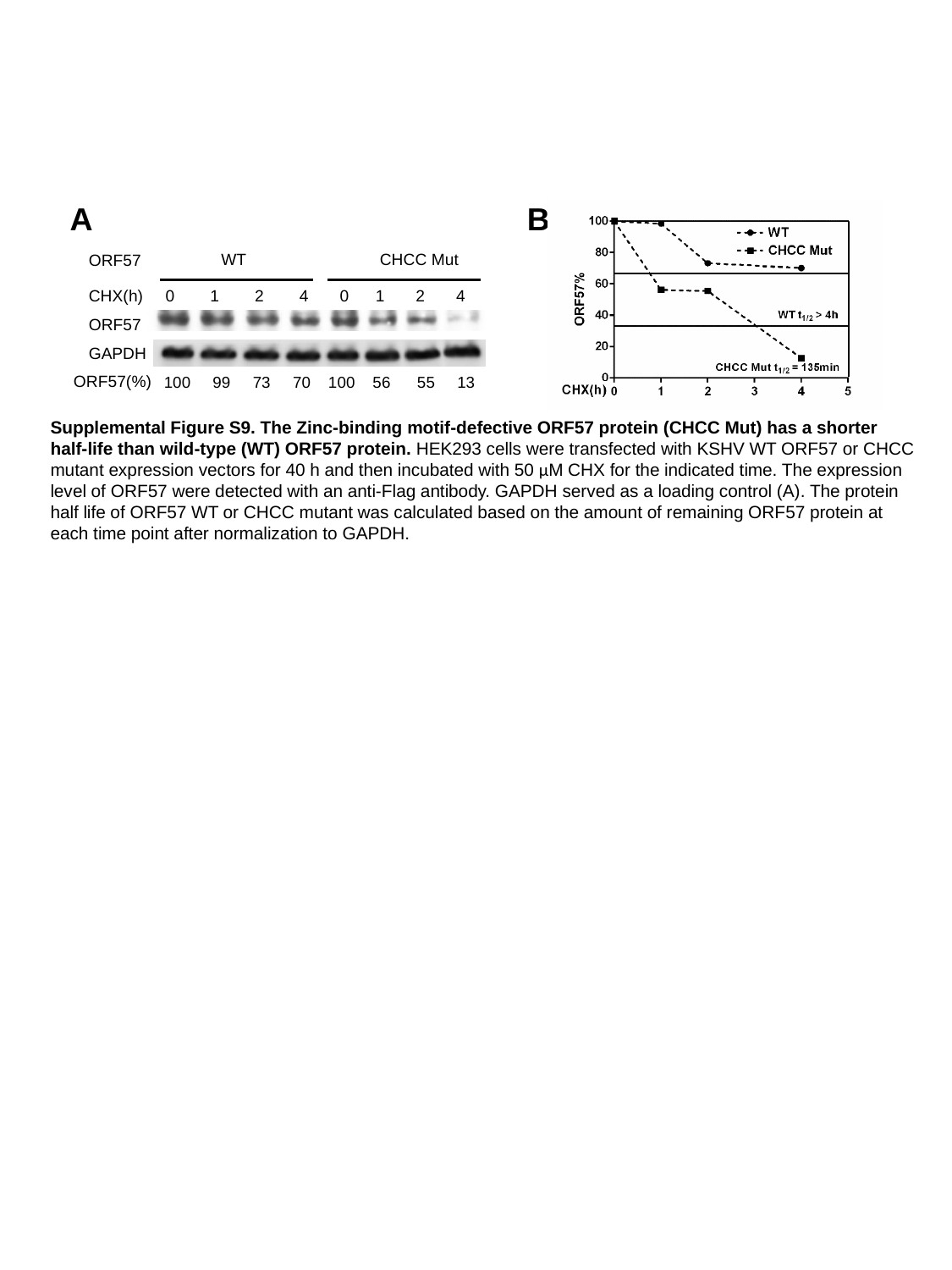

A
B
WT
CHCC Mut
ORF57
CHX(h) 0 1 2 4 0 1 2 4
ORF57
GAPDH
ORF57(%)
100 99 73 70 100 56 55 13
Supplemental Figure S9. The Zinc-binding motif-defective ORF57 protein (CHCC Mut) has a shorter half-life than wild-type (WT) ORF57 protein. HEK293 cells were transfected with KSHV WT ORF57 or CHCC mutant expression vectors for 40 h and then incubated with 50 μM CHX for the indicated time. The expression level of ORF57 were detected with an anti-Flag antibody. GAPDH served as a loading control (A). The protein half life of ORF57 WT or CHCC mutant was calculated based on the amount of remaining ORF57 protein at each time point after normalization to GAPDH.
