# Supplementary material for: The crystal structure of KSHV ORF57 reveals dimeric active sites important for protein stability and function
Source: PLoS Pathog. 2018 Aug 10;14(8):e1007232. doi: 10.1371/journal.ppat.1007232 (PMC6105031; doi:10.1371/journal.ppat.1007232)
Supplement: S10 Fig — The genome of the mutant virus (C333S/H423L/C427S/C432S) was constructed by two step scarless recombination in BAC16. (A) Sequencing results of the recombinant BAC16 confirmed the introduced four mutations. (B) The integrity of recombinant BAC16 was digested with XhoI and the digestion products were separated on a 0.8% agarose gel (lane 1: WT; lane 2: CHCC mutant; lane 3: 1 Kb DNA ladder). The DNA bands bearing ORF57 segments are marked with red triangles. (PPTX) [file ppat.1007232.s010.pptx]

## Slide 1
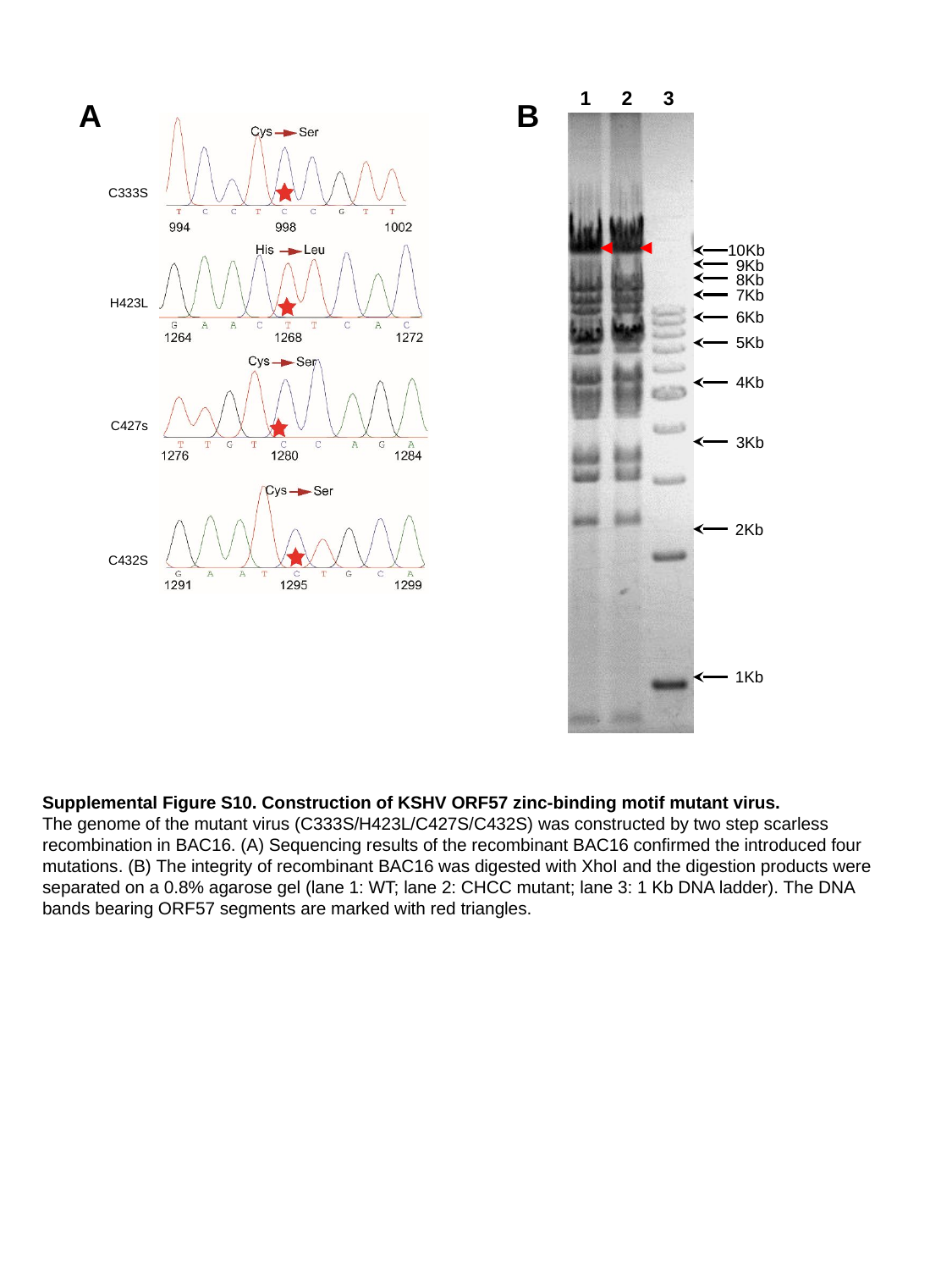

1
2
3
A
B
C333S
10Kb
9Kb
8Kb
7Kb
H423L
6Kb
5Kb
4Kb
C427s
3Kb
2Kb
C432S
1Kb
Supplemental Figure S10. Construction of KSHV ORF57 zinc-binding motif mutant virus.
The genome of the mutant virus (C333S/H423L/C427S/C432S) was constructed by two step scarless recombination in BAC16. (A) Sequencing results of the recombinant BAC16 confirmed the introduced four mutations. (B) The integrity of recombinant BAC16 was digested with XhoI and the digestion products were separated on a 0.8% agarose gel (lane 1: WT; lane 2: CHCC mutant; lane 3: 1 Kb DNA ladder). The DNA bands bearing ORF57 segments are marked with red triangles.
