# Supplementary material for: The crystal structure of KSHV ORF57 reveals dimeric active sites important for protein stability and function
Source: PLoS Pathog. 2018 Aug 10;14(8):e1007232. doi: 10.1371/journal.ppat.1007232 (PMC6105031; doi:10.1371/journal.ppat.1007232)
Supplement: S11 Fig — (A) The α-helix 4 (yellow) inserts into the core region of the globular domain (left two panels). The residues E287, E288, W292 in α-helix 4 mediate a large number of interactions (black dashed lines) with the surrounding residues (highlighted in cyans), leading to structure stabilization (right panel). (B) The residue K345 protrudes out from the surface of ORF57-CTD and does not form any interaction with other residues. (PPTX) [file ppat.1007232.s011.pptx]

## Slide 1
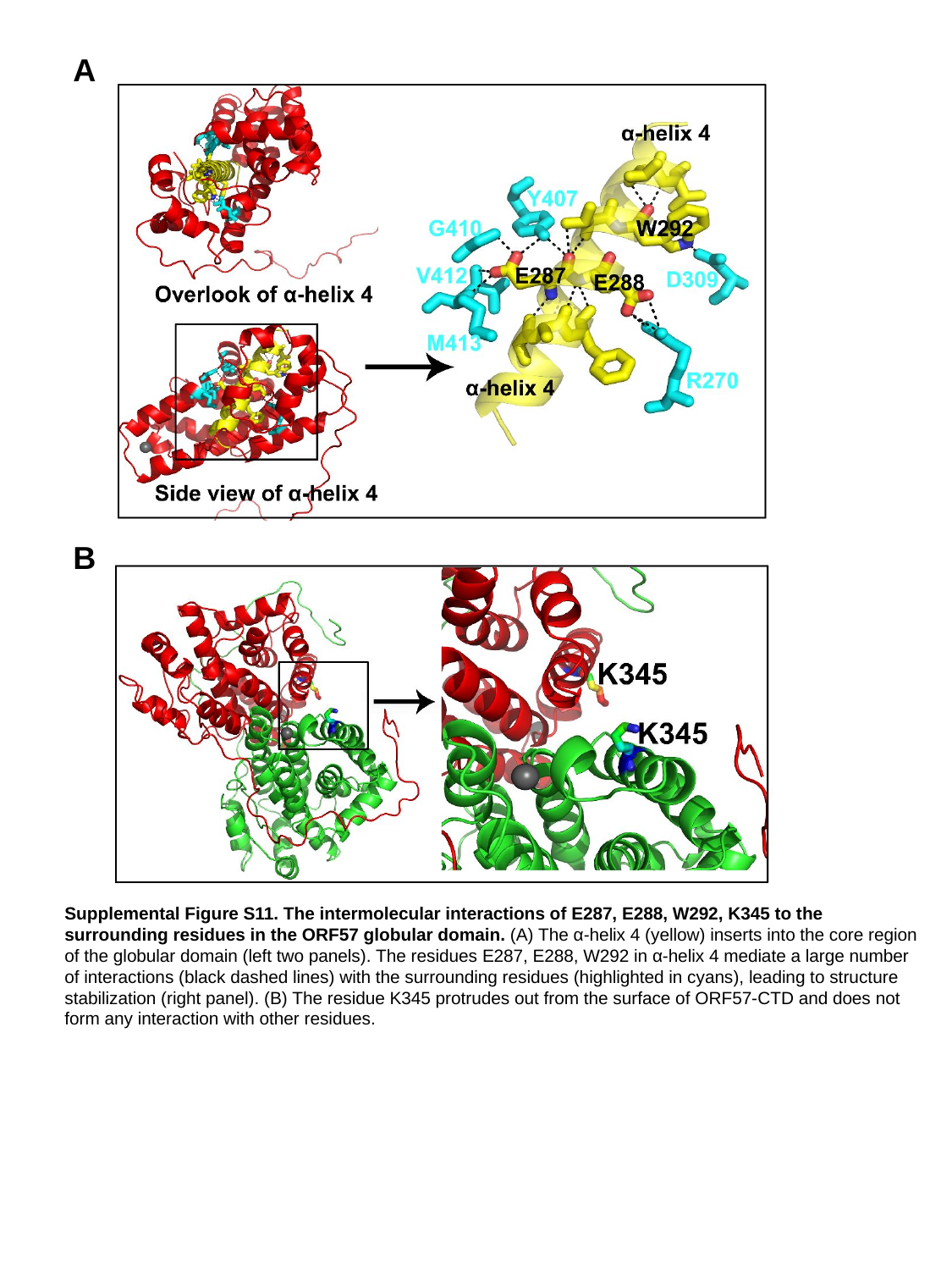

A
B
Supplemental Figure S11. The intermolecular interactions of E287, E288, W292, K345 to the surrounding residues in the ORF57 globular domain. (A) The α-helix 4 (yellow) inserts into the core region of the globular domain (left two panels). The residues E287, E288, W292 in α-helix 4 mediate a large number of interactions (black dashed lines) with the surrounding residues (highlighted in cyans), leading to structure stabilization (right panel). (B) The residue K345 protrudes out from the surface of ORF57-CTD and does not form any interaction with other residues.
