# Supplementary material for: The crystal structure of KSHV ORF57 reveals dimeric active sites important for protein stability and function
Source: PLoS Pathog. 2018 Aug 10;14(8):e1007232. doi: 10.1371/journal.ppat.1007232 (PMC6105031; doi:10.1371/journal.ppat.1007232)
Supplement: S12 Fig — (A and B) The shape and electrostatic calculations of a monomeric structure in dimerized ORF57-CTD (A) and ICP27-CTD (B, PDB ID: 4yxp) calculated by PyMol with the APBS with180˚ rotation, red, negative charge; blue, positive charge. (PPTX) [file ppat.1007232.s012.pptx]

## Slide 1
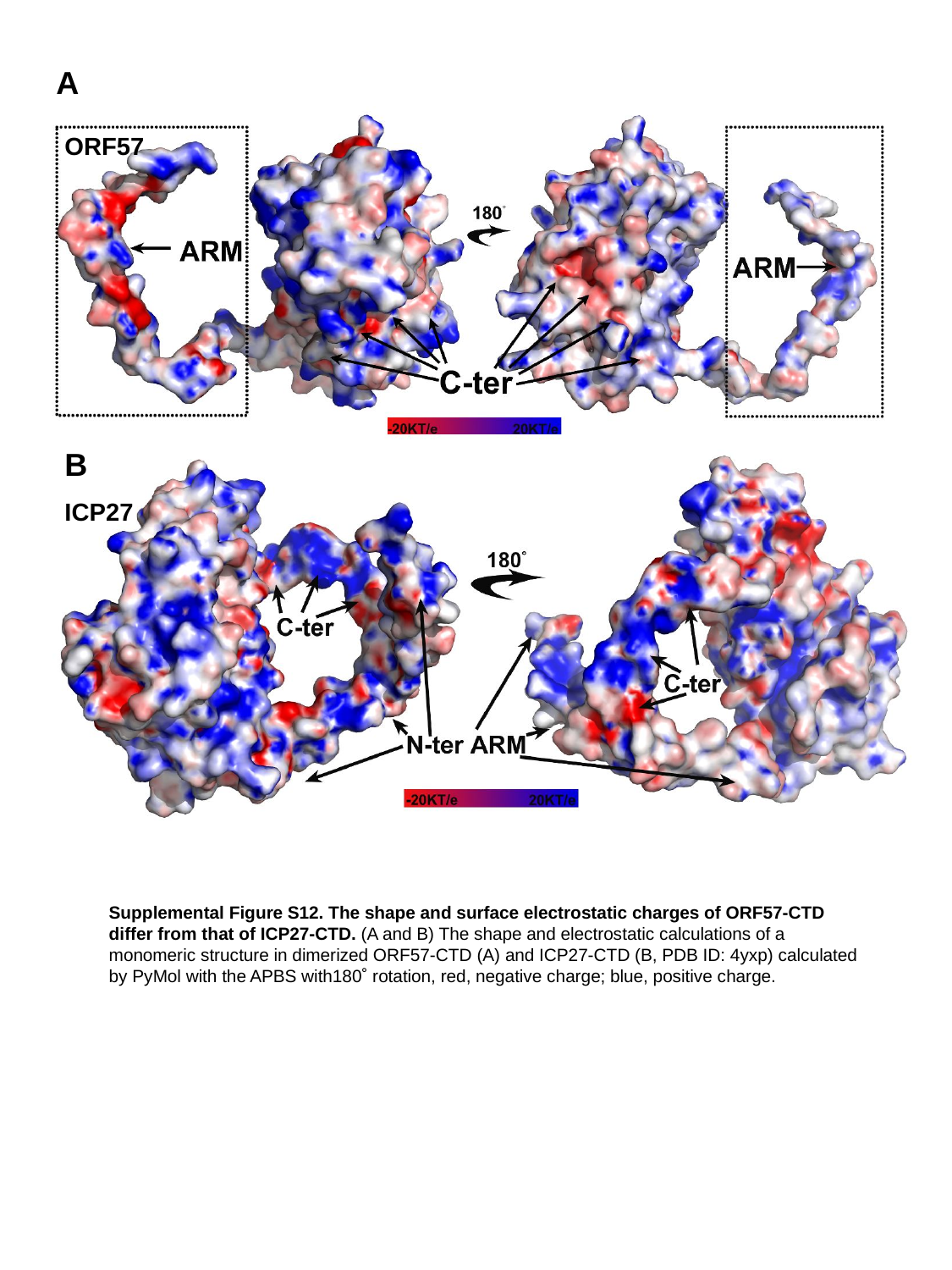

A
ORF57
B
ICP27
Supplemental Figure S12. The shape and surface electrostatic charges of ORF57-CTD differ from that of ICP27-CTD. (A and B) The shape and electrostatic calculations of a monomeric structure in dimerized ORF57-CTD (A) and ICP27-CTD (B, PDB ID: 4yxp) calculated by PyMol with the APBS with180˚ rotation, red, negative charge; blue, positive charge.
