# Supplementary material for: The crystal structure of KSHV ORF57 reveals dimeric active sites important for protein stability and function
Source: PLoS Pathog. 2018 Aug 10;14(8):e1007232. doi: 10.1371/journal.ppat.1007232 (PMC6105031; doi:10.1371/journal.ppat.1007232)
Supplement: S1 Table — (DOCX) [file ppat.1007232.s015.docx]

Supplemental Table 1 Cloning primers and Northern probe sequences

| ORF57-Δ167-219-FLAG | GATGACGACGTCAGACAGGACAGGGATATCACCGCT |
| --- | --- |
|  | AGCGGTGATATCCCTGTCCTGTCTGACGTCGTCATC |
| ORF57-Δ219-FLAG | ACTCAGAATTCACCATGGACAGGGATATCACCGCTCTC |
|  | ATCGTGGATCCAGAAAGTGGATAAAAGAATAAACCCTTG |
| ORF57-Δ166-GFP | TACTCAGAATTCACCATGGGTGTGTCTGACGCCGTAAAG |
|  | ATCGTGGATCCGAAAGTGGATAAAAGAATAAACCCTTG |
| ORF57-Δ219-GFP | ACTCAGAATTCACCATGGACAGGGATATCACCGCTCTC |
|  | ATCGTGGATCCGAAAGTGGATAAAAGAATAAACCCTTG |
| ORF57-5’ss-GFP | CTGGGTTTCTTGCCGTCGAGAAACCGCAGC |
|  | GACGGCAAGAAACCCAGTACGCTTGATTGT |
| ORF57-FLAG | TACTCAGAATTCACCATGGTACAAGCAATGATAGACATGG |
|  | ATCGTGGATCCAGAAAGTGGATAAAAGAATAAACCCTTG |
| ORF57-GFP | TACTCAGAATTCACCATGGTACAAGCAATGATAGACATGG |
|  | ATCGTGGATCCGAAAGTGGATAAAAGAATAAACCCTTG |
| ORF57-R270A/R271A-FLAG | AGTTGCGCTGCAGAAACCGCAGCCGCCGGA |
|  | GGTTTCTGCAGCGCAACTCACCCAGTACGC |
| ORF57-R325A/R327A-FLAG | GTTACCGCATTTGCATTACTTCATCTTTCC |
|  | AAGTAATGCAAATGCGGTAACAAACGCATT |
| ORF57-HA | CCGGAATTCATGGTACAAGCAATGATAGACATGG |
|  | CGCGGATCCAGAAAGTGGATAAAAGAATAAACCC |
| ORF57-C333S-HA | TACTTCATCTTTCCTCCGTTTTTGACAAGCAGAGC |
|  | GCTCTGCTTGTCAAAAACGGAGGAAAGATGAAGTA |
| ORF57-H423L-HA | GTGATAGTAATGGAACTTCACAGCTTGTGCAG |
|  | CTGCACAAGCTGTGAAGTTCCATTACTATCAC |
| ORF57-C427S-HA | GAACATCACAGCTTGTCCAGAAACAGTGAATGTG |
|  | CACATTCACTGTTTCTGGACAAGCTGTGATGTTC |
| ORF57-C432S-HA | GCAGAAACAGTGAATCTGCAGCGGCAACCCGG |
|  | CCGGGTTGCCGCTGCAGATTCACTGTTTCTGC |
| ICP27-HA | CCGGAATTCATGGCGACTGACATTGATATGC |
|  | CCGCTCGAGAAACAGGGAGTTGCAATAAAAATAT |
| ICP27-C400S-HA | CGGCCCTTTCTCCAGTCCTACCTGAAGGCGCGA |
|  | TCGCGCCTTCAGGTAGGACTGGAGAAAGGGCCG |
| ICP27-H479L-HA | GAAATCCTAGACACACTCCGCCAGGAGTGTTCG |
|  | CGAACACTCCTGGCGGAGTGTGTCTAGGATTTC |
| ICP27-C483S-HA | ACACACCGCCAGGAGTCTTCGAGTCGTGTCTGC |
|  | GCAGACACGACTCGAAGACTCCTGGCGGTGTGT |
| ICP27-C488S-HA | TGTTCGAGTCGTGTCTCCGAGTTGACGGCCAGT |
|  | ACTGGCCGTCAACTCGGAGACACGACTCGAACA |
| EB2-HA | CCCAAGCTTATGGTTCCTTCTCAGAGACT |
|  | TGCTCTAGATTGATTTAATCCAGGAACA |
| EB2-C354S-HA | CAAAGAGCTGGCACCCTCCTTCCTTCCTAACAC |
|  | GTGTTAGGAAGGAAGGAGGGTGCCAGCTCTTTG |
| EB2-H445L-HA | AGGCCGTGGAGACTCTCACACGTGACTGCCG |
|  | CGGCAGTCACGTGTGAGAGTCTCCACGGCCT |
| EB2-C449S-HA | ACTCACACACGTGACTCCCGAAGTGCATCATGC |
|  | GCATGATGCACTTCGGGAGTCACGTGTGTGAGT |
| EB2-C454S-HA | TGCCGAAGTGCATCATCCAGCCGACTTGTCAGG |
|  | CCTGACAAGTCGGCTGGATGATGCACTTCGGCA |
| UL69-HA | CGCGGATCCATGGAGCTGCACTCACGC |
|  | CCGGAATTCGTCATCCATATCATCGCTGT |
| UL69-C387S-HA | AACCTGGACCTGGGCTCCATCCTGGACCACCAG |
|  | CTGGTGGTCCAGGATGGAGCCCAGGTCCAGGTT |
| UL69-H494LS-HA | GGCGCCCTGCAGTGCCTCGAGTGTCAGAACGAG |
|  | CTCGTTCTGACACTCGAGGCACTGCAGGGCGCC |
| UL69-C496S-HA | CTGCAGTGCCACGAGTCTCAGAACGAGATGTGC |
|  | GCACATCTCGTTCTGAGACTCGTGGCACTGCAG |
| UL69-C501S-HA | TGTCAGAACGAGATGTCCGAACTGCGCATCCAA |
|  | TTGGATGCGCAGTTCGGACATCTCGTTCTGACA |
| mORF57-HA | CCGGAATTCATGGCACAGCAGATGTTGGA |
|  | CCGCTCGAGTTCACACACAAAAAATACCCCC |
| mORF57-C289S-HA | ATAGAGGTGATAAAATCTATAGAAAAGGATGGA |
|  | TCCATCCTTTTCTATAGATTTTATCACCTCTAT |
| mORF57-H378L-HA | GCTGCACTAAATTGTCTTCAGTGTAACAAAGAC |
|  | GTCTTTGTTACACTGAAGACAATTTAGTGCAGC |
| mORF57-C380S-HA | CTAAATTGTCATCAGTCTAACAAAGACTGTGAT |
|  | ATCACAGTCTTTGTTAGACTGATGACAATTTAG |
| mORF57-C384S-HA | CAGTGTAACAAAGACTCTGATAAATGTAAATAT |
|  | ATATTTACATTTATCAGAGTCTTTGTTACACTG |
| mORF57-C387S-HA | AAAGACTGTGATAAATCTAAATATATTTTGGAT |
|  | ATCCAAAATATATTTAGATTTATCACAGTCTTT |
| Northern blot probe | GGGCACTGGAGTGGCAAC |
