# Supplementary material for: The crystal structure of KSHV ORF57 reveals dimeric active sites important for protein stability and function
Source: PLoS Pathog. 2018 Aug 10;14(8):e1007232. doi: 10.1371/journal.ppat.1007232 (PMC6105031; doi:10.1371/journal.ppat.1007232)
Supplement: S2 Table — (DOCX) [file ppat.1007232.s016.docx]

Supplemental Table 2.BAC16 mutagenesis primers

| FIRST ROUND UP: | 5’GGGGATGTGATGGGGCTACTAAACGTGATAGTAATGGAACTTCACAGCTTGTCCAGAAACAGGATGACGACGATAAGTAGGG3’ |
| --- | --- |
| FIRST ROUND DOWN: | 5’GTTGCCGCTGCACATTCACTGTTTCTGGACAAGCTGTGAAGTTCCATTACTATCACGTTTCAACCAATTAACCAATTCTGATTAG3’ |
| SECOND ROUND UP: | 5’ATAGTAATGGAACTTCACAGCTTGTCCAGAAACAGTGAATCTGCAGCGGCAACCCGGGCCAGGATGACGACGATAAGTAGGG3’ |
| SECOND ROUND DOWN: | 5’AATTTGGCCGACCCCATTGCGGCCCGGGTTGCCGCTGCAGATTCACTGTTTCTGGACAAGCAACCAATTAACCAATTCTGATTAG3’ |
| THIRD ROUND UP: | 5’AATGCGTTTGTTACCAGATTTAGATTACTTCATCTTTCCTCCGTTTTTGACAAGCAGAGCAGGATGACGACGATAAGTAGGG3’ |
| THIRD ROUND DOWN: | 5’CTGTTTGATCAGTGCTAGCTGCTCTGCTTGTCAAAAACGGAGGAAAGATGAAGTAATCTACAACCAATTAACCAATTCTGATTAG3’ |
