# Supplementary material for: The crystal structure of KSHV ORF57 reveals dimeric active sites important for protein stability and function
Source: PLoS Pathog. 2018 Aug 10;14(8):e1007232. doi: 10.1371/journal.ppat.1007232 (PMC6105031; doi:10.1371/journal.ppat.1007232)
Supplement: S3 Table — (DOCX) [file ppat.1007232.s017.docx]

Supplemental Table 3. qPCR primers

| GAPDH UP: | TACTAGCGGTTTTACGGGCG |
| --- | --- |
| GAPDH DOWN: | TCGAACAGGAGGAGCAGAGAGCGA |
| K8.1 UP: | AAAGCGTCCAGGCCACCACAGA |
| K8.1 DOWN: | GGCAGAAAATGGCACACGGTTAC |
| ORF59 UP: | TTGGCACTCCAACGAAATATTAGAA |
| ORF59 DOWN: | CCCCTGGCCTGTAGTATTCCA |
| ORF57 UP: | TGGCGAGGTCAAGCTTAACTTC |
| ORF57 DOWN: | CCCCTGGCCTGTAGTATTCCA |
| mORF57 UP: | GTAGGGAGTTGCCCACTGAC |
| mORF57 DOWN: | CCACGTAAGAACTGGGCCTT |
| EB2 UP: | TACAACCCTGGCACGCTAAG |
| EB2 DOWN: | AGAATGGCCCTGACAAGTCG |
| UL69 UP: | GTTCGTCGTCAATAAGCGCC |
| UL69 DOWN: | ACATAGGGCGTCCTCCTCTT |
| K9 UP: | GGCCCACTAATATGTCAGCCA |
| K9 DOWN: | CATTGTCCCGCAACCAGACT |
