# Supplementary material for: The crystal structure of KSHV ORF57 reveals dimeric active sites important for protein stability and function
Source: PLoS Pathog. 2018 Aug 10;14(8):e1007232. doi: 10.1371/journal.ppat.1007232 (PMC6105031; doi:10.1371/journal.ppat.1007232)
Supplement: S4 Table — (DOCX) [file ppat.1007232.s018.docx]

Supplemental Table 4 PDBePISA analysis of interactions in the dimer interface

| Hydrogen bonds(ARM-Globular) | | | |
| --- | --- | --- | --- |
|  | Structure A | Distance [Å] | Structure B |
| 1 | VAL 367[N] | 3.12 | ASP 184[OD1] |
| 2 | ARG 372[NE] | 3.42 | ILE 194[O] |
| 3 | TYR 452[OH] | 2.54 | CYS 202 [O] |
| 4 | GLN 280[NE2] | 3.60 | VAL 205[O] |
| 5 | ARG 437[NE2] | 2.51 | PRO 208[O] |
| 6 | ARG 437[NE] | 3.6 | ALA209 [O] |
| 7 | VAL 205[N] | 2.59 | GLN 280 [OE1] |
| 8 | LEU 175[N] | 2.58 | GLU 366[OE2] |
| 9 | ASP 184[N] | 3.72 | GLU 366[O] |
| 10 | SER 187[OG] | 3.43 | GLU 398[OE1] |
| 11 | ARG 189 [NH2] | 3.81 | LEU 399[O] |
| 12 | ARG 189 [NH2] | 3.12 | THR 402[OG1] |
| 13 | ARG 197 [NH2] | 3.47 | TYR 406[O] |
| 14 | ARG 197 [NH1] | 3.49 | ARG 408[O] |
| 15 | ARG 197 [NH1] | 2.84 | ASP 411[OD1] |
| 16 | ARG 197 [NH1] | 2.73 | ASP 411[OD2] |
| 17 | PHE 206[N] | 3.15 | ASN 417[OD1] |
| 18 | ASP 184 [OD1] | 3.49 | ARG 395[NH1] |
| 19 | ILE 194[O] | 3.48 | ARG 372 [NE] |
| 20 | ILE 194[O] | 3.87 | ARG 372 [NH2] |
| 21 | CYS 202[SG] | 3.46 | ASN 417[ND2] |
| 22 | CYS 202[O] | 2.87 | TYR 452[OH] |
| 23 | ALA 209 [O] | 3.49 | ARG 437 [NH1] |
| 24 | THR 402 [OG1] | 3.27 | ARG 189[NH2] |
| 25 | TYR 406[O] | 3.59 | ARG 197[NH2] |
| 26 | ASP 411[OD1] | 2.67 | ARG 197[NH1] |
| 27 | ASP 411[OD2] | 2.71 | ARG 197[NH1] |
| 28 | ASN 417[OD1] | 3.41 | PHE 206[N] |
| Hydrogen bonds(Globular -Globular) | | | |
| 1 | TYR 349[OH] | 2.23 | TYR 349[OH] |
| 2 | ARG 327[NH2] | 3.7 | ALA 352[O] |
| 3 | ARG 327[NH2] | 3.65 | MET 353[SD] |
| 4 | ALA 275 [O] | 3.58 | ARG 325 [NH1] |
| 5 | ALA 352 [O] | 2.86 | ARG 327 [NH2] |

| Salt bridges(ARM-Globular) | | | |
| --- | --- | --- | --- |
|  | Structure A | Distance [Å] | Structure B |
| 1 | LEU 175[N] | 3.54 | GLU 366[OE1] |
| 2 | LEU 175[N] | 2.58 | GLU 366[OE2] |
| 3 | ARG 197[NH1] | 2.84 | ASP 411[OD1] |
| 4 | ARG 197[NE] | 3.40 | ASP 411[OD1] |
| 5 | ARG 197[NH1] | 2.73 | ASP 411[OD2] |
| 6 | ARG 197[NH2] | 3.74 | ASP 411[OD2] |
| 7 | ASP 184[OD1] | 3.49 | ARG 395 [NH1] |
| 8 | ASP 411[OD1] | 2.67 | ARG 197 [NH1] |
| 9 | ASP 411[OD2] | 2.71 | ARG 197 [NH1] |
| 10 | ASP 411[OD2] | 2.88 | ARG 197 [NH2] |
| Salt bridges (Globular -Globular) | | | |
| 1 | GLU 431[OE2] | 3.61 | ARG271 [NH2] |
